# Stress-induced IL-6 regulation in pediatric bone growth disorders: current insights and therapeutic strategies

**DOI:** 10.3389/fendo.2026.1833900

**Published:** 2026-06-02

**Authors:** Zaineb Sohail, Norhayati binti Abd. Hadi, Azizur Rehman, Muhammad Asghar, Farasat Zaman

**Affiliations:** 1 Faculty of Health Sciences, Universiti Sultan Zainal Abidin – UniSZA, Terengganu, Malaysia; 2 Department of Life Sciences, School of Science, University of Management and Technology, Lahore, Pakistan; 3 Halal Research Centre, Universiti Sultan Zainal Abidin- UniSZA, Terengganu, Malaysia; 4 Trustlife Laboratories, Drug Research & Development Center, Istanbul, Türkiye; 5 Department of Biomedical Engineering, School of Mechanical and Manufacturing Engineering (SMME), National University of Sciences & Technology, National University of Sciences and Technology (NUST), Islamabad, Pakistan; 6 Department of Biology, Lund University Sweden, Lund, Sweden; 7 Department of Sports Sciences and Clinical Biomechanics, University of Southern Denmark, Odens, Denmark; 8 Faculties of Rehabilitation & Allied Health Sciences, Health & Medical Sciences and Pharmaceutical Sciences, Riphah International University, Islamabad, Pakistan; 9 Department of Women’s and Children’s Health, Karolinska Institutet & University Hospital, Stockholm, Sweden; 10 Division of Pediatric Endocrinology, Karolinska University Hospital, Stockholm, Sweden

**Keywords:** BMI, bone growth, children, diet, IL-6, inflammation, nutrition, stress

## Abstract

Stress-induced upregulation of interleukin-6 (IL-6) signaling and its downstream pathophysiological consequences have garnered considerable attention in recent years. However, no comprehensive review has specifically examined the association between stress-induced IL-6 and its implications for bone health in children. During childhood, linear growth and peak bone mass acquisition are tightly regulated processes. Hence, this review aims to investigate current evidence on stress-induced IL-6 upregulation and its detrimental effects on pediatric bone health. Findings from animal models, knockout studies, pediatric inflammatory disorders, including juvenile idiopathic arthritis, pediatric systemic lupus erythematosus and inflammatory bowel disease, and models of metabolic stress collectively demonstrate that stress triggers IL-6, thereby impairing skeletal growth and increasing fragility. Data shows that persistent IL-6 upregulation not only disrupts the normal functioning of growth hormone, insulin-like growth factor-1 (GH/IGF-1) axis, enhances receptor activator of nuclear factor kappa-B ligand (RANKL)-mediated osteoclastogenesis, and promotes bone marrow adiposity. Further, stress-induced high levels of IL-6 adversely affect the skeletal, immune, and endocrine systems, thereby compromising skeletal development and bone growth in children. Elevated systemic or local IL-6 levels may exert direct deleterious effects on bone by impairing stem cell differentiation and inhibiting the proliferation and maturation of growth plate chondrocytes, ultimately restricting longitudinal bone growth. Moreover, elevated IL-6 levels may also impair muscle health and crosstalk between muscle and bone, thereby compromising skeletal integrity. Collectively, these findings underscore the need for integrative therapeutic strategies that target inflammation and redox imbalance in bone and other tissues, particularly in children. Better understanding stress-induced IL-6 dysregulation is critical for pediatric bone development and long-term skeletal health.

## 1 Introduction

**Figure 1:**
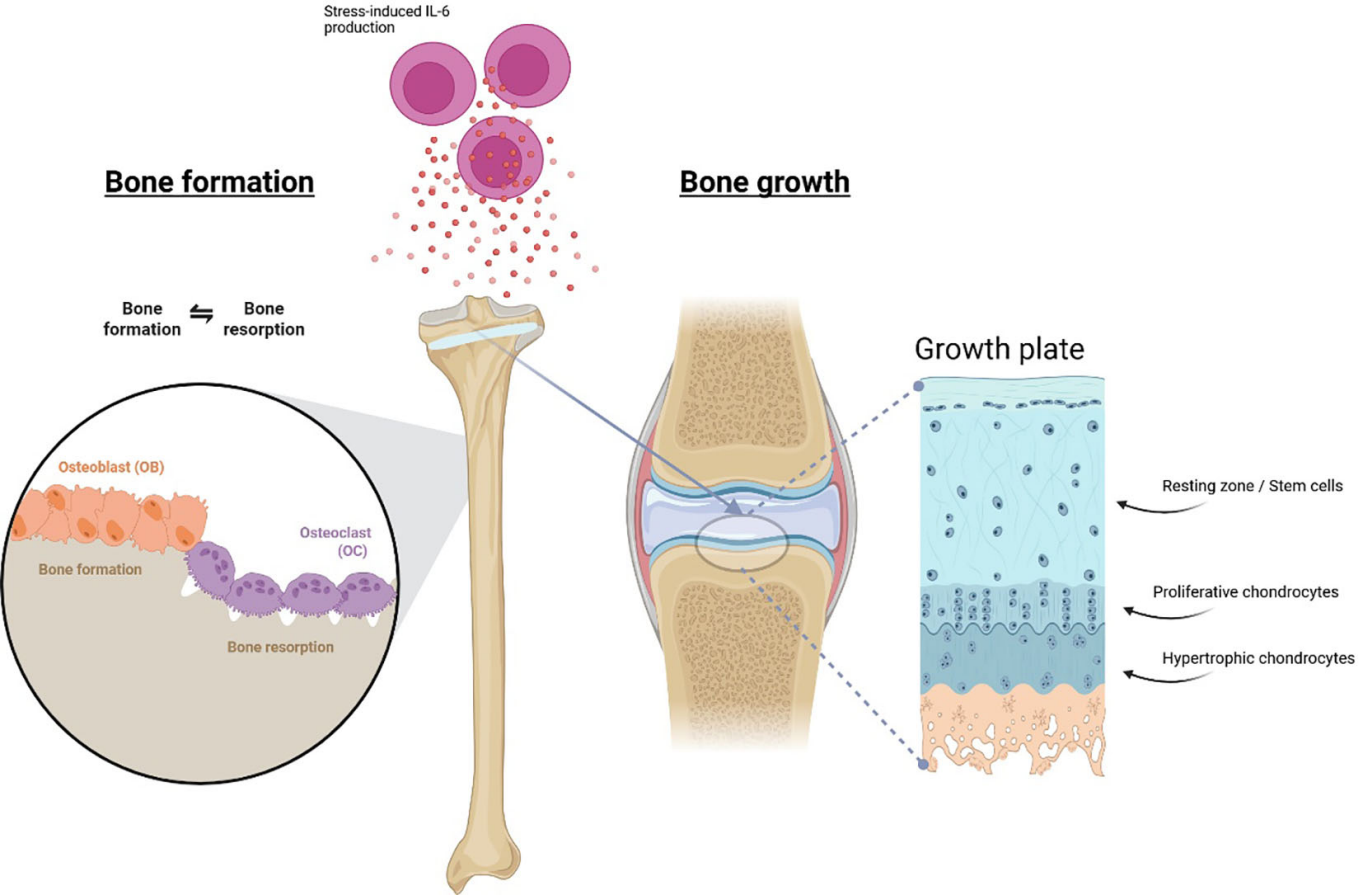
Illustration of a long bone structure, showing osteoblasts, osteoclasts and chondrocytes. Growth plate cartilage consists of resting zone chondrocytes (also known as stem cells), proliferative chondrocytes, and hypertrophic chondrocytes. Stress-induced IL-6 production negatively affects both osteoblasts and growth plate chondrocytes, thereby decreasing bone formation and longitudinal bone growth. IL-6 can suppress the proliferation of chondrocytes and their differentiation within the growth plate cartilage, thereby impairing chondrogenesis.

In children and adolescents, bone health mainly reflects two interconnected processes: one, longitudinal growth, which is facilitated by chondrocyte proliferation in the growth plate, and the other, bone formation (regulated by osteoblasts/osteoclasts activity), as depicted in **Figure 1** (1). These processes are meticulously governed by nutritional, immunological, and endocrine elements that jointly dictate linear growth as well as skeletal strength. Any disruption in these regulatory pathways may affect bone mineral density and skeletal fragility, ultimately leading to growth retardation. Interleukin-6 (IL-6) is a proinflammatory cytokine that affects endochondral ossification at the growth plate level by impairing chondrocyte proliferation, differentiation, and hypertrophy through multiple signaling pathways, including phosphoinositide 3-kinase/protein kinase B (PI3K/AKT, Janus kinase/signal transducer and activator of transcription 3 (JAK/STAT3) and mitogen-activated protein kinase (MAPK). This leads to disruption of growth plate organization and ultimately reduced longitudinal bone growth in pediatric populations and experimental models (2–4). Similarly, studies in children showed that psychosocial stress activates neuroendocrine pathways, including the Hypothalamic Pituitary Adrenal (HPA) axis and sympathetic system, thereby promoting inflammatory signaling and elevating circulating IL-6 levels during childhood and adolescence (5–9). A recent comprehensive review also reported that psychological stress induces both acute and chronic inflammatory responses, leading to increased reactive oxygen species (ROS) generation, which may adversely affect bone health in children (10). In adults, psychological stress increases IL-6 production in multiple cell types including adipocytes, monocytes/macrophages, endothelial cells and osteoblast-lineage cells through neuroendocrine pathways, particularly the HPA axis and sympathetic signaling (11–14). Psychological stress may also elevate IL-6 levels via non-immune cells specifically adipocytes (15). An intricate yet complex role of brown adipose tissue (BAT), which is also known as brown fat or good fat, was explored by Qing et al. (16) in murine models in which stress induced IL-6 production from BAT; although it helped in dealing with stress but at the cost of the body’s immune strength due to activation of the inflammatory cascade. Experimental studies in adult murine bone cells demonstrate that IL-6 signaling in osteoblasts and osteocytes enhances osteoclast differentiation through RANKL-dependent pathways (17). Psychological stress does not act through single cell but it affects multiple cells and elevates the cell-specific IL-6 which converges to amplify systemic inflammation with downstream consequences as compromised immune health or bone fragility (18). In pediatric bone disorders, increased levels of inflammatory cytokines, including IL-6, have been linked to changes in bone metabolism and lower bone density, suggesting that inflammatory signaling may shift bone remodeling towards increased resorption (19).

When IL-6 is chronically or repetitively triggered, it elevates the inflammatory pathway and worsens diseased conditions in the body (13). Not only this, it provides an acceleratory push to osteoclastogenesis, which mediates bone degeneration and promotes several bone-related pathological conditions (20). In pediatric rheumatic disease, control of inflammation with biologics, targeting IL-6 pathway, is associated with improved growth trajectories, supporting a contributory role for elevated IL-6 activity in growth impairment (21, 22). However, direct pediatric data identifying IL-6 as the sole causative factor for osteoporosis or fractures is scarce; the majority of evidence is indirect and originates from conditions characterized by chronic inflammation e.g., Inflammatory Bowel Disease (IBD) and Juvenile Idiopathic Arthritis (JIA), where IL-6 levels increase in conjunction with other cytokines and risk modifiers such as disease activity, corticosteroids, malnutrition and immobility (23, 24).

Consistent with this, current definitions and diagnostic approaches to pediatric osteoporosis emphasize its multifactorial etiology rather than attribution to a single cytokine (25).

Recent investigations consistently associate chronic stress exposure with increased IL-6 levels in childhood and young adults, especially when stress coincides with metabolic risk (26). Elevated IL-6 and soluble IL-6 receptor (sIL-6R) levels are associated with low bone mineral density (BMD) and altered bone remodeling in adult populations (27, 28). In pediatric inflammatory diseases such as JIA, chronic systemic inflammation has similarly been linked to impaired bone accrual and reduced bone density during growth (29). Given its central role at the interface between immune regulation and skeletal homeostasis, IL-6 represents both a biomarker of skeletal risk and a potential therapeutic target.

The evidence reviewed in this manuscript is largely associative rather than causal. The available pediatric data are predominantly derived from studies of chronic inflammatory diseases, like IBD and JIA in which elevated IL-6 co-exists with multiple confounders including disease activity, corticosteroid use, malnutrition, and reduced mobility. This narrative review, therefore, examines the association between stress and IL-6 regulation, with particular emphasis on how stress-induced IL-6 upregulation may adversely affect bone health during growth, a relatively underexplored aspect of pediatric disease biology.

## 2 Methodology

A structured literature search was performed for this narrative review using PubMed, Web of Science, Scopus, and Google Scholar databases, which includes studies published up to October 2025. The keywords and MeSH headings were used as “IL-6, “ “stress” (psychological, oxidative, HPA axis), “bone growth, “ “growth plate, “ “osteoblast, “ “osteoclast, “ “mesenchymal stem cells, “ and pediatric conditions such as juvenile idiopathic arthritis, systemic lupus erythematosus, inflammatory bowel disease, and rickets. Therapeutic terms including “IL-6 blockade, “ “Tocilizumab (TCZ), “ and “trans-signaling inhibition” were also incorporated. Search terms like “nutrient adequacy and inflammatory cytokines”, “micronutrient deficiency”, “phytochemical + IL-6”, “diet and stress”, “BMI and IL-6” were also used to find publications demonstrating the effect of nutrient adequacy on stress and inflammation. Boolean operators were applied to refine searches.

Studies that met the criteria included peer-reviewed original research on human, pediatric, and animal subjects; mechanistic *in vitro* studies; randomized controlled trials (RCTs) targeting IL-6; pediatric studies based on observations linking IL-6 to bone growth/health; and subsequent high-quality reviews providing mechanistic insights. Studies such as non-English publications, conference abstracts that did not provide full data, and studies not relevant to bone biology, stress physiology, or IL-6 signaling were excluded. A narrative methodology was selected to connect mechanistic, preclinical, and clinical strategies into a cohesive conceptual framework connecting stress biology to pediatric skeletal development.

## 3 IL-6 signaling

In 1986, Kishimoto and Hirano in 1986 first identified and cloned IL-6 as a cytokine that has potential to modulate B-cell development (30). It comprises of glycoprotein (~21–28 kDa) and is produced by the immune cells like T-cells, monocytes/macrophages and activated synovial fibroblasts, endothelial cells, keratinocytes, mesangial cells, stromal cells, adipocytes and bone-related cells like osteoblasts in response to any stressed condition (infection, tissue injury, or sterile inflammation) (31, 32).

### 3.1 Receptors and signaling modes: classic (cis) vs trans signaling

**Figure 2:**
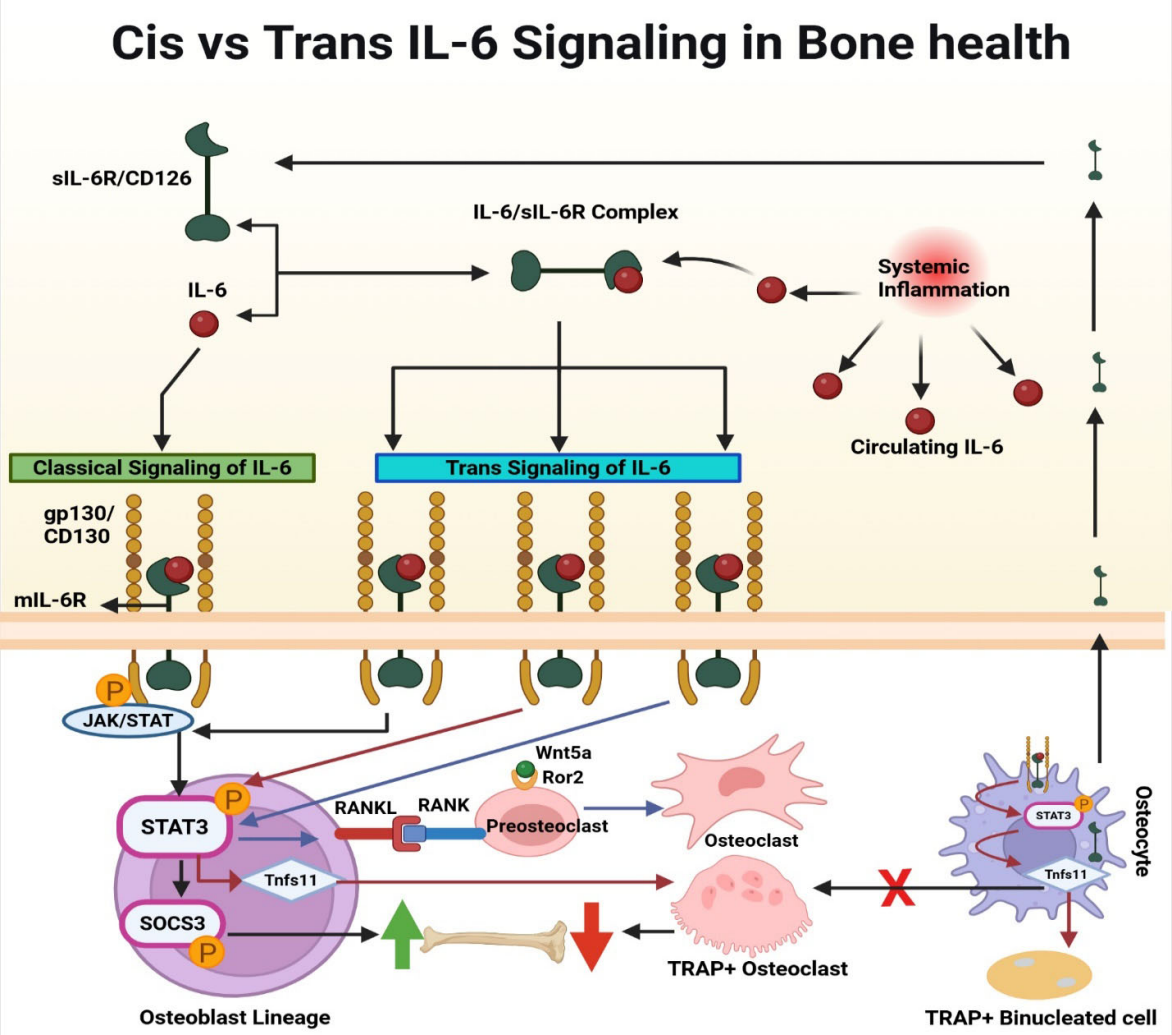
Cis- versus trans-IL-6 signaling pathways in bone remodeling and osteoimmune regulation. The figure illustrates the diverse roles of classical (cis) and trans IL-6 signaling in bone remodeling and bone health. In classical signaling of IL-6, IL-6 binds to the IL-6 receptor which is membrane bound (mIL-6R/CD126), thus leading to gp130 (CD130) dimerization that leads to the activation of downstream JAK/STAT3 signaling cascade. This signaling cascade promotes osteoblast survival and enhances regulatory feedback through SOCS3. In contrast, trans signaling of IL-6 is initiated when circulating IL-6 molecules form a complex with soluble form of IL-6R enabling the activation of gp130 on cells that lack mIL-6R. This mechanism extends towards downstream signaling by inducing the inflammatory response during systemic inflammation and enhances STAT3 phosphorylation and favors osteoclastogenesis through increased RANKL–RANK interactions and pre-osteoclast maturation into mature osteoclasts via the modulation of Tnfs11 expression, thereby influencing osteoclast differentiation. This IL-6/sIL-6R complex also induces the TRAP-positive multinucleated osteoclasts. Overall, this figure illustrates the importance of cis and trans signaling of IL-6 that explains how the balance between cis and trans IL-6 signaling potentially determines the shift between bone formation and resorption (bone remodeling).

IL-6 signaling acts through a two-component receptor system, as shown in **Figure 2**; IL-6 initially associates with the IL-6 receptor (IL-6R/CD126), subsequently recruiting gp130 (CD130) to establish a high-affinity signaling complex (32). Classic (cis) signaling necessitates membrane-bound IL-6R and is confined to IL-6R-positive cells (e.g., hepatocytes, specific leukocytes), whereas trans signaling emerges when IL-6 associates with sIL-6R and this complex then activates ubiquitous gp130 on nearly all cell types, significantly exponentiating the cellular scope of IL-6 activity (32, 33).

Classic signaling is generally associated with regenerative and homeostatic responses, whereas trans signaling predominantly promotes inflammatory pathways and contributes to disease pathogenesis, providing a rationale for selective inhibition of the trans-signaling route (34–36). The IL-6 gene is swiftly activated by Nuclear Factor kappa-light-chain-enhancer of activated B cells (NF-κB) and AP-1 (Activator Protein-1) following the stimulation of pattern-recognition and cytokine receptors, while context-specific post-transcriptional mechanisms regulate the intensity and duration of its expression (32, 33). IL-6 signals through a receptor complex consisting of IL-6 receptor subunits (IL-6R; CD126) and gp130 (CD130), facilitating signal transduction into cells (37). Circulating IL-6 concentrations rise sharply during systemic inflammation and may stay persistently increased during inflammation; however, sIL-6R and soluble gp130 (sgp130) together regulate and spatially constrain IL-6 signaling in the circulation (33, 38).

IL-6R can exist in both forms: a soluble form that lacks membrane-binding sites and a membrane-bound form (mIL-6R). sIL-6R has a tendency to make receptor complexes with gp130 thus, transduces downward signaling cascade in response to IL-6, analogous to mIL-6R (39). This distinctive signaling mechanism, termed trans-signaling, possesses attributes that diverge from those of conventional cytokine signaling pathways, such as TNF-α, where the soluble receptor acts as a decoy receptor (40). Notably, elevated concentrations of sIL-6R can persist during convalescence and in chronic inflammatory conditions, suggesting that the potential for IL-6 trans-signaling may extend beyond the acute inflammatory phase (41). IL-6 signaling is well characterized in experimental and adult studies and is conserved across ages; however, age-related differences in immune regulation and cytokine responsiveness may vary in receptor expression and intracellular feedback remain poorly understood in pediatric populations (42, 43).

### 3.2 Downstream pathways (JAK/STAT3, MAPK, PI3K/AKT, SOCS)

Following IL-6/gp130 interaction, Janus kinases (JAK1, JAK2, or Tyrosine Kinase 2 also known as TYK2) are activated, which results in the gp130 phosphorylation and the recruitment of STAT3. Subsequently, dimerization and translocation of STAT3 takes place towards the nucleus, where it manages genes related to proliferation, differentiation, survival, and metabolism (44). Experimental studies in mouse embryonic fibroblast models demonstrate that IL-6 signaling can enhance osteogenic differentiation through STAT3-dependent pathways and downstream mTORC1 activation (45). Furthermore, IL-6 signaling cascade activates the MAPK (ERK1/2, p38) and PI3K/AKT pathways. The MAPK pathway controls cellular proliferation and matrix synthesis, whereas PI3K/AKT regulates cell survival, and growth factors sensitivity (46). IL-6 augments BMP9-induced osteogenesis via the STAT3/mTORC1 signaling pathway in mesenchymal stem cells (45). Negative feedback is regulated by Suppressors of Cytokine Signaling (SOCS) proteins, specifically SOCS3 (44). Disruption of any of these pathways in bone, like hyper-STAT3 or over activation of PI3K/AKT, may cause aberrant bone remodeling, decreased chondrogenesis and poor growth plate maturation, or osteoporosis (47).

### 3.3 IL-6 regulation in bone

The modulation of IL-6 signaling in the skeletal system is triggered by multiple layers of control. Endogenous inhibitors such as soluble gp130 (sgp130) function as decoy receptors for the IL-6/sIL-6R complex, thereby specifically obstructing trans-signaling while preserving classic signaling (48). IL-6 receptor blockade with a Monoclonal antibody (MR16-1) against the IL-6 receptor improved bone healing and preserved trabecular structure in adolescent mice with ischemic osteonecrosis, highlighting therapeutic potential (49). Tumor Necrosis Factor alpha (TNF-α) and Interleukin-1 beta (IL-1β) stimulate IL-6 production in osteoblasts, whereas IGF-1 and growth hormone signaling can counteract IL-6–driven bone resorption (50). Moreover, IL-6 regulates the RANKL/Osteoprotegerin (OPG) axis by enhancing RANKL expression and lowering OPG secretion, hence shifting the equilibrium towards osteoclast activation and bone resorption in conditions when it is persistently raised (45). This regulatory network is particularly important in growth plate cartilage and longitudinal growth: persistent high levels of IL-6 may halt chondrocyte hypertrophy, that may results in diminished long-bone growth (51, 52).

### 3.4 Effect of stress on IL-6 regulation

In children, psychological stress is rapidly recognized as a potential factor for dysregulating immune responses, thus, influencing disease susceptibility (53). Psychological or environmental stressors trigger the HPA axis and sympathetic nervous system (SNS), leading to transient increases in circulating IL-6 (54). In contrast, chronic stress is associated with higher expression of IL-6 and C-reactive protein (CRP), consistent with persistent low-grade inflammation (5). Interestingly, in children and adolescents, perceived or caregiver-related stress correlates positively with circulating and salivary IL-6 concentrations (55).

In adult subjects, the IL-6 response to acute stress follows a delayed kinetic profile, with minimal change in the first 10 minutes, a marked rise at 40–50 minutes, and peak concentrations at 90–120 minutes (56). This time frame is the reason that many experimental frameworks studying IL-6 measure its levels repeatedly after stressor’s encounter (57). Mechanistically, acute stress engages sympathetic β-adrenergic pathways that induce IL-6 release; studies in animal models identify brown adipose tissue as a key non-immune source, supporting a role for IL-6 as a systemic stress-responsive cytokine.

In adult humans and animal models, chronic stress promotes a sustained pro-inflammatory phenotype through altered glucocorticoid (GC) sensitivity and dysregulated β-adrenergic signaling alongside persistently elevated peripheral inflammatory markers (58–60). Similarly, a longitudinal study in adolescents found that early exposure to a harsh family environment is associated with a persistent pro-inflammatory state with reduced GC sensitivity, alternation in β-adrenergic signaling, and increased inflammatory markers (61). Longitudinal human studies, including caregiving cohorts, demonstrate progressive increases in IL-6 over time, linking chronic stress to accelerated inflammatory ageing (62). At the genomic level, chronic stress is associated with reduced GC receptor signaling and enhanced NF-κB–mediated transcription, a profile that favors heightened IL-6 responsiveness to subsequent challenges (60). Preclinical murine models of social stress, similarly, show amplified IL-6 response, where early IL-6 elevations predict subsequent stress susceptibility; pharmacological attenuation of IL-6 in these settings has been linked to improved skeletal outcomes (63). Moreover, preclinical data demonstrates that stress-induced IL-6 can trigger peripheral myeloid cells and accelerate downstream neuroimmune and behavioral effects (64).

In **Table 1**, types of stress/oxidative stimuli are discussed and how they increase IL-6 across species and tissues.

**Table 1:** Stress-induced IL-6 elevation and its relevance to bone growth in pediatric and juvenile models. .

Type of stress/stimulus	Species	Tissue/cell source	Effect on IL-6	Functional outcome	Bone-specific outcomes	Age group/model	Relevance to pediatric bone health	Ref.
Acute psychosocial stress (public speaking + mental arithmetic; Trier Social Stress Test for Children)	Human	Peripheral blood plasma	IL-6 rises significantly after stress;	Innate immune activation; HPA axis and sympathetic nervous system stimulation	No direct bone measurement. Elevated IL-6 promotes bone resorption and reduces bone formation via RANKL/OPG imbalance	Adolescents (mean age 13.9 years; N = 84)	High, shows real-time IL-6 rise from stress specifically in adolescents, the key window for building peak bone mass	(65)
Cumulative childhood/adolescent stress, maltreatment, poverty, interpersonal adversity (meta-analysis of 187 studies, 922 effect sizes)	Human	Peripheral blood (IL-6, CRP, TNF-α)	Childhood stress consistently linked to higher IL-6; association grows stronger with age	Chronic low-grade inflammation; immune system programming; biological embedding of early adversity	Sustained IL-6 elevation during the bone accrual years suppresses GH/IGF-1 axis and promotes osteoclastogenesis, reducing peak bone mass	Children and adolescents (0–18 years)	Very high, broadest evidence base linking childhood stress to IL-6; covers the entire pediatric bone-growth period	(5)
Chronic inflammatory disease (Juvenile Idiopathic Arthritis/Inflammatory Bowel Disease)	Human	Serum (systemic IL-6); growth plate chondrocytes (local effects)	Persistently elevated serum IL-6, IL-1β, and TNF-α; IL-6 blocks GH receptor signalling and IGF-1 production via SOCS pathway	GH/IGF-1 axis disrupted; chondrocyte proliferation impaired; pubertal delay	Direct bone outcomes: short stature (10–41% of JIA patients); reduced bone mineral density; impaired growth plate function; reduced trabecular bone volume	Children with JIA or IBD (pediatric clinical + *in vitro* studies)	Highest bone relevance, directly proves IL-6 elevation causes growth failure and reduced bone mass in children	(66)
Chronic mild stress (CMS), repeated mild stressors (cage tilt, wet bedding, restraint, disrupted lighting, social isolation) over 4 weeks	Mouse	Bone tissue (femur, vertebra); serum corticosterone; bone norepinephrine	Corticosterone and bone norepinephrine markedly elevated; IL-6 pathway activated via sympathetic nervous system	SNS stimulation reduces osteoblast number; increases osteoclast activity; net bone loss	Direct: significant trabecular and cortical bone loss; reduced bone formation rate; osteoblast numbers fall. All reversed by antidepressant (imipramine) or propranolol, proving causal pathway	Adult male mice (12 weeks old; 4–5 week CMS	High mechanistic evidence, establishes causal stress that cause SNS/IL-6 increase, bone loss pathway; mechanistically analogous to stress in children and adolescents	(67)
Chronic psychosocial/social defeat stress (chronic subordinate colony housing; repeated social subordination over 19 days)	Mouse	Plasma (systemic IL-6); bone marrow and growth plate (sympathetic innervation markers)	Sympathetic innervation at the growth plate increased (↑TH+ cells in bone marrow); HPA axis dysregulated	Growth plate cartilage-to-bone transition disrupted; Runx2+ chondrocyte differentiation altered;	shortened femur and tibia length; widened growth plate; dysregulated endochondral ossification, direct proof that psychosocial stress impairs linear bone growth	Adolescent/juvenile mice (7 weeks old; adolescent equivalent) 19-day CSC	Very high, one of very few studies using an adolescent mouse model showing psychosocial stress directly stunts bone growth via growth plate disruption	(68)
Neonatal inflammatory stress, lipopolysaccharide (LPS) injection in rat pups on postnatal days 3 and 5	Rat	Systemic blood (IL-6, IL-1β acute response); brain (prefrontal cortex oxidative stress at juvenile stage P40)	Neonatal LPS triggers acute IL-6 and IL-1β surge; leads to persistent oxidative stress (reduced glutathione) in the prefrontal cortex at the juvenile stage (postnatal day 40)	Early-life immune activation programs neuroimmune dysregulation into the juvenile period; oxidative stress persists into adolescence; social behaviour disrupted	No direct bone measurement. Oxidative stress at P40 (juvenile stage) occurs during rapid bone growth, conditions known to impair osteoblast function and bone mineralisation	Neonatal rats (P3, P5) assessed at juvenile P40 and adult P70 (Sprague-Dawley)	High, models neonatal infection/sepsis-like stress with IL-6 surge and lasting oxidative effects during the juvenile bone growth phase; translates to premature infants and early-childhood illness	(69)
Prenatal maternal immune activation (MIA), poly(I:C) injection during pregnancy (gestational day E12.5; viral-mimetic activating maternal immune response)	Mouse	Maternal serum; placenta; fetal brain and gene expression	IL-6 identified as the causal mediator: a single IL-6 injection replicates all MIA effects; blocking IL-6 with antibody or IL-6 knockout prevents all behavioural deficits; IL-6 suppresses fetal IGF-1	Fetal neurodevelopmental reprogramming; schizophrenia/autism-like behaviours in offspring; IGF-1 suppressed during critical prenatal growth window	No direct bone measurement. IL-6-driven IGF-1 suppression during fetal development is a plausible mechanism for impaired skeletal programming in offspring	Pregnant mice (E12.5); outcomes assessed in adult offspring (prenatal stress model)	Moderate, establishes IL-6 as the key prenatal stress mediator;	(70)

This table summarizes how different stressors increase IL-6 and highlights associated functional and bone-specific outcomes. It distinguishes between pediatric, juvenile, and adult models and indicates the translational relevance to pediatric bone health, noting that direct bone evidence remains limited in many studies. IL-6, interleukin-6; OPG, osteoprotegerin; GH, growth hormone; IGF-1, insulin-like growth factor-1; JIA, juvenile idiopathic arthritis; RANKL, receptor activator of nuclear factor κB ligand; HPA axis, hypothalamic–pituitary–adrenal axis; IBD, inflammatory bowel disease; SNS, sympathetic nervous system; CMS, chronic mild stress; MIA, maternal immune activation;.

Apart from psychological stress, oxidative stress (OS) also acts as a potent upstream IL-6 regulator.

Excess ROS that is generated during any stressful situation, like mitochondrial dysfunction, inflammation, or environmental stress exposure, leads to the activation of redox-sensitive transcription factors directly, including NF-κB and MAPK pathways, thus leading to IL-6 upregulation across immune and non-immune cell types. Chronic oxidative stress therefore retains IL-6 signaling cascade, reinforcing low-grade inflammation and tissue remodeling processes, which are relevant to growth, metabolism, and skeletal homeostasis.

### 3.5 Inflammation, obesity, and marrow adiposity by stress induced IL-6

Obesity and chronic low-grade inflammation (characterized by elevated IL-6) are shown to be associated with increased bone-marrow adipose tissue (BMAT) and decreased BMD, thereby endorsing an imbalance in bone marrow adipose tissue that holds clinical significance for both adults and at-risk youth (71). The apposition of marrow adiposity under inflammatory or metabolic stress is associated with less bone formation, mechanistically aligning with IL-6–induced lineage shifts reported in preclinical studies (72).

### 3.6 Effects of IL-6 on mesenchymal and bone-marrow mesenchymal stromal cells

BMAT is a dynamic endocrine organ that expands in response to obesity, aging and chronic inflammation. The proliferation of BMAT is often associated with reduced bone density and osteogenic activity in both pediatric and adult populations, which depicts a competitive relationship between adipogenesis and osteogenesis in the bone marrow microenvironment (73, 74). IL-6 is a key regulator of this lineage balance. Under metabolic stress, systemic and local IL-6 elevations promote commitment of bone marrow stromal cells (BMSCs) towards adipogenesis via STAT3/Peroxisome Proliferator-Activated Receptor Gamma (PPARγ) signaling. Under metabolic stress, systemic and local IL-6 elevations promote commitment of BMSCs towards adipogenesis via STAT3–PPARγ signaling (75, 76).

Consistently, Li et al. also demonstrated that IL-6-deficient mice exposed to a high-fat diet retain osteogenic capacity, exhibit minimal marrow adiposity and maintain expression of osteogenic transcription factors, including Runt-related transcription factor 2 (RUNX2) and osterix, compared with wild-type controls (77). Thus, these findings further support the role of IL-6 in suppressing osteoblastogenesis while promoting adipogenesis under metabolic stress.

Clinical and experimental data further link sustained high levels of IL-6 in obesity, juvenile idiopathic arthritis (JIA), and type 2 diabetes to increased bone-marrow fat fraction and reduced bone formation (78–81). In murine models, bone-marrow adipocytes and osteoblasts derive from a common mesenchymal progenitor. It is observed that sustained IL-6 exposure shifts osteogenic progenitors toward an adipogenic lineage by inhibiting the Wnt/β-catenin pathway (82).

This competition between lineages is specifically bad during childhood and adolescence, a time period when bone modeling and linear growth are highly active (83). Prolonged high levels of IL-6 observed in pediatric obesity or chronic inflammatory conditions, lead to the premature depletion of osteoprogenitor reserves, increased marrow adiposity, and impaired bone accrual (77, 84–86). These effects together cause a decrease in peak bone mass, thereby predisposing to skeletal fragility later in life.

On the other hand, anti-IL-6 receptor drugs, such as like TCZ or metabolic methods used for blocking IL-6 signaling, which lower systemic inflammation, have been associated with restoration of osteogenic markers and attenuation of marrow fat expansion in adult and pediatric studies (87–92). Collectively, these observations position IL-6 as a molecular switch linking inflammation and metabolism to skeletal remodeling.

#### 3.6.1 Proliferation & survival of MSCs under IL-6 Influence

Bone marrow-derived mesenchymal stem cells (BM-MSCs) are known as multipotent stromal cells and the main origin of osteoblasts (93). IL-6 can exert differential effects on MSC proliferation and differentiation. Under defined conditions, IL-6 can enhance MSC proliferation and promote osteogenic commitment via autocrine and paracrine STAT3 signaling. For example, Bone Morphogenetic Protein 9 (BMP9)-induced osteogenic differentiation is accompanied by hugh IL-6 expression, which contributes to upregulation of osteogenic markers and matrix mineralization (45). However, these effects are highly context-specific, and IL-6 may also inhibit alternative lineage differentiation, including adipogenic and chondrogenic pathways, depending on the signaling milieu (4).

#### 3.6.2 Effects of IL-6 on osteoblasts

Osteoblasts not only produce IL-6 but also respond to the IL-6 signaling, and this makes IL-6 one of the key communication molecules in bone remodeling. Expression of IL-6R increases during osteoblast differentiation and IL-6 exerts stage-dependent effects, hence in earlier stages, it promotes osteoblast differentiation marker expression while in mature osteoblasts it leads to apoptosis under certain conditions (94). IL-6 interacts with major osteoblast differentiation pathways.

Experimental models indicate that IL-6 enhances RUNX2 and alkaline phosphatase expression and supports mineral deposition in specific contexts, such as BMP9-driven systems (45). Nonetheless, prolonged exposure to increased IL-6 may adversely affect osteoblast maturation (95, 96), although direct preclinical studies in growth-plate or long-bone models are scarce. Modulation of osteoclast formation is the major downstream consequence of osteoblast-lineage-IL-6 as it promotes osteoclastogenesis indirectly by osteoblast/stromal cell pathways, while it also shows context-dependent direct effects on osteoclast precursors as seen *in vitro* (40, 97). In line with this functional significance, bone nodule formation can be inhibited by IL-6 exposure (98), suggesting that the net outcome of IL-6 on osteoblasts is highly context-dependent and differentiation-stage dependent.

IL-6 interfaces with key osteogenic pathways, including BMP–SMAD and Wnt–β-catenin signaling, although its effects remain context-dependent (99). In some models, IL-6 suppresses osteoblastogenesis and matrix mineralization by downregulating RUNX2, Osterix (OSX), and Osteocalcin (OCN) (2), whereas in others it enhances osteogenic activity and matrix formation (100, 101).

### 3.7 Effects on osteoclastogenesis & resorption

IL-6 promotes bone resorption through both indirect and direct mechanisms. Indirectly, IL-6 stimulates osteoblast-lineage and stromal cells to increase RANKL expression and suppress OPG, thereby shifting the RANKL/OPG ratio in favor of osteoclastogenesis (102, 103). IL-6 signaling also accelerates RANKL expression while suppressing OPG in extreme stress or inflammatory conditions, thus increasing the RANKL/OPG ratio, favoring osteoclast precursor differentiation and activation (104, 105). This paracrine mechanism does not need the direct IL-6 action on osteoclast but rather depends on osteoblast-lineage cells to interpret inflammatory signals into increased bone resorption (106). Thus bone remodeling shifts towards bone resorption in the presence of sustained increased IL-6 expression, leading to loss of bone and compromised skeletal integrity, especially in chronic inflammation (107).

Directly, IL-6 may act on osteoclast precursor cells (108). IL-6 stimulated osteoclastogenesis despite the presence of OPG, indicating a RANKL-independent mechanism and highlighting a direct pro-resorptive action (109, 110). This dual pathway (indirect via RANKL and direct on precursors) underscores IL-6’s capacity to modulate bone resorption in inflammatory states. IL-6 trans-signaling via sIL-6R activates the JAK/STAT3 and MAPK pathways in monocyte/macrophage lineage cells, thereby enhancing precursor survival, differentiation potential, and resorptive function (40, 111). IL-6 alone cannot fully induce osteoclast differentiation; however, it significantly enhances RANKL and M-CSF signaling, thereby accelerating osteoclastogenesis in chronic inflammatory or stress-related conditions (97, 112).

Although the osteoclastogenic effects of IL-6 are well characterized in adult human studies and experimental models, direct mechanistic evidence in pediatric bone cells remains limited. Nevertheless, clinical data from pediatric inflammatory conditions provide important indirect support for a similar biological role during skeletal growth (19).

### 3.8 Effects of IL-6 on growth plate chondrocytes and longitudinal bone growth

The growth plate is the primary location for longitudinal bone formation, and clinical findings show that chondrocyte proliferation, differentiation, and matrix remodelling are significantly influenced by IL-6 (113). The IL-6 utilizes the gp130 receptor to activate the JAK/STAT3 pathway, which is crucial for sustaining chondrocyte proliferation and survival in the proliferative zone as demonstrated in the murine model (postnatal mice) (114). Under physiological settings, the temporary activation of STAT3 by IL-6 facilitates endochondral ossification and the turnover of cartilage matrix (115).

Nonetheless, under chronic inflammation, sustained exposure to IL-6 disturbs this equilibrium. Long-term exposure to IL-6 not only inhibits the expression of Insulin-like growth factor-1 (IGF-1) but also diminishes the production of type II and type X collagen, thus halting the hypertrophic differentiation and extracellular matrix synthesis (116–118). In mouse models of systemic inflammation and obesity, high levels of IL-6 have been reported with reduced tibial length, reduced hypertrophic zone, and disorganized chondrocyte columns, illustrating its suppressive impact on longitudinal bone growth (77). Similarly, high IL-6 levels can halt the growth by reducing IGF-1 levels, highlighting how chronic inflammation is linked to IGF-1 and thus contributes to growth impairment (51). IL-6–mediated inflammatory signaling may also interact with endocrine stress responses. Increased IL-6 levels can enhance GC sensitivity in osteoblasts and growth-plate chondrocytes, thereby amplifying growth inhibition under chronic stress conditions (119, 120). Experimental studies further suggest that maternal or chronic social stress can induce IL-6 overexpression and delay bone formation in offspring, highlighting the potential long-term skeletal consequences of cytokine-mediated stress responses during early development (26, 51).

Importantly, pharmacological inhibition of IL-6 signaling has demonstrated the potential to reverse several of these abnormalities. Experimental treatment with IL-6 pathway inhibitors such as TCZ or soluble gp130-Fc reduces osteoclast infiltration in inflammatory models (49). Altogether, these findings suggest that IL-6 is a critical mediator linking systemic inflammation to impaired endochondral bone formation and highlights its potential as a therapeutic target to prevent growth retardation in chronic inflammation.

## 4 IL-6 and ROS signaling in bone pathophysiology

### 4.1 Stress-induced ROS production

Psychological stress triggers the sustained release of GCs and catecholamines via the activation of the HPA axis SNS (sympathetic nervous system) that disrupts mitochondrial electron transport and stimulates Nicotinamide Adenine Dinucleotide Phosphate (NADPH) oxidase, thus promotes excessive ROS generation (121, 122). When antioxidant responses are compromised, stress-induced ROS accumulation undermines cellular redox equilibrium, accelerates lipid peroxidation and DNA deterioration, and amplifies redox-sensitive inflammatory signaling pathways, including NF-κB (123). Thus, as a result, oxidative stress induced by chronic stress acts as a vital mechanistic connection between psychosocial stress exposure and inflammation-related tissue dysfunction, including less development and bone remodeling (124).

### 4.2 ROS-induced IL-6 regulation and osteoclast activation

In adult experimental and pediatric models, oxidative stress is known to be a potent upstream driver of IL-6 regulation within the bone microenvironment (47, 125, 126). In adult rheumatoid arthritis models, redox-sensitive transcription factors, including NF-κB and AP-1 are activated under high ROS conditions and subsequently increase IL-6 transcription in osteoblasts, stromal cells, and bone-marrow progenitors (127). Increased IL-6 levels trigger the pro-resorptive milieu by inhibiting OPG and concurrently elevating RANKL expression, thus altering the RANKL/OPG ratio in favor of osteoclastogenesis in adult experimental and inflammatory disease models (102). Whether this RANKL/OPG imbalance is similarly operative in the pediatric bone microenvironment during active growth phases requires further direct study. This imbalance actively drives the differentiation and activation of osteoclast precursors, leading to increased bone resorption and microarchitectural degradation. Oxidative stress expedites bone loss predominantly via IL-6–dependent STAT3 and NF-κB signaling pathways, which promote both RANKL expression and osteoclast viability (95).

Overall, these data suggest that ROS and IL-6 act in a positive feedback loop, where oxidative stress triggers IL-6 production, and IL-6, in turn, enhances ROS production through inflammatory signaling. This synergistic interaction forms a mechanistic basis by which oxidative stress contributes to poor bone health, especially in chronic inflammatory conditions.

### 4.3 Oxidative stress in pediatric bone growth

Children with chronic inflammatory diseases or metabolic stress often exhibit elevated IL-6 levels and increased oxidative stress. Both chronic inflammation and metabolic stress are well known for their negative effects on bone health, especially during phases of rapid growth in pediatric population (128). In children with JIA, elevated levels of IL-6 increase production of free radicals and reduced antioxidant capacity, which contributes to reduced chondrocyte proliferation, differentiation and bone mineralization (126, 129).

Similarly, metabolic stress state in pediatric patients with obesity is known to trigger mitochondrial dysfunction, high ROS generation, and low-grade chronic inflammation along with high IL-6 levels (78). Children with obesity exhibit increased marrow adiposity and reduced osteogenic differentiation, outcomes strongly linked to IL-6–driven oxidative signaling pathways (79). High levels of ROS not only trigger IL-6 but also directly halt osteoblast function and growth-plate maturation. Because childhood and adolescence are periods of high bone turnover and rapid longitudinal bone growth, the combined burden of oxidative stress and elevated IL-6 can have long-term negative consequences for peak bone mass and bone growth.

In summary, chronic pediatric diseases with chronic inflammation or metabolic dysfunction create a ROS-IL-6 pathological loop that compromises bone formation, linear growth, and skeletal strength. A better understanding of this link is necessary for developing new treatment strategies to protect bone health in children exposed to persistent physiological or psychosocial stressors.

As it is already mentioned that the HPA axis and SNS get activated by stress, either psychological or physiological, and this not only enhances ROS levels but also induces IL-6 from various cells like immune cells, adipose tissues, skeletal muscles and bone-related cells through GC and Catecholamine- dependent pathways (130, 131). Sustained stress-induced IL-6 levels can promote low-grade inflammation that disturbs normal growth by interfering with the GH–IGF-1 axis as well as growth plate chondrocyte function, as demonstrated in IL-6 transgenic mouse models (132) and supported by reviews of pediatric inflammatory disease data (116, 133). Thus, chronic stress induced IL-6 levels represent a plausible connection between psychological stress and impaired linear growth especially during the critical pediatric growth phase (134).

#### 4.3.1 Stress-induced IL-6 regulation, bone metabolism and microarchitecture

In addition to developmental delay, stress-induced elevation of IL-6 facilitates osteoclastogenesis through the RANKL/OPG pathway, diminishes trabecular bone volume, and disrupts calcium homeostasis (95). Children and adolescents exposed to early life adversity or maltreatment show elevated circulating inflammatory markers including IL-6 (128). Although this association suggests a potential pathway linking stress to compromised skeletal health, direct primary studies measuring bone mineral density (BMD) z-scores alongside IL-6 levels in stressed pediatric populations are needed to confirm this relationship. Collectively, it is suggested that psychosocial stress and inflammatory signaling provide a physiologic connection between emotional adversity and compromised skeletal growth.

### 4.4 Animal models/knockout studies/overexpression models

#### 4.4.1 Knockout models (Il6−/−)

In murine high-fat diet-induced metabolic stress, no trabecular deterioration and marrow-fat expansion were observed in IL-6 knockout mice (135, 136). Moreover, osteogenic marker expression was retained in them in comparison to wild-type controls, which indicates that IL-6 is a potent driver of bone fragility and adipogenic drift under a high-fat diet (HFD) (77, 136).

#### 4.4.2 Cell-specific IL-6 signaling in osteoblasts

In mouse models, osteoblast-targeted genetics revealed that IL-6 signaling in osteoblasts is essential for the high-turnover adaptation to aerobic exercise; disruption of this pathway diminished formation indices *in vivo*, underscoring that physiological (cis) signaling can be anabolic in a context- and time-dependent manner (113, 137).

#### 4.4.3 Overexpression/transgenic IL-6

In IL-6 transgenic mice designed to model chronic pediatric inflammation, show a striking skeletal phenotype with impaired growth, comprising reduced trabecular mass, high osteoclast number (138). This, therefore, is early, yet relevant proof that persistent IL-6 high levels are catabolic to the skeletal integrity.

#### 4.4.4 Trans-signaling (IL-6/sIL-6R/gp130)

In adult female mouse models mimicking postmenopausal estrogen deficiency, IL-6 trans-signaling has been associated with trabecular bone loss following ovariectomy; inhibiting trans-signaling maintained trabecular bone in that animal model (139). Subsequently, it is indicated that context is significant: in a mucolipidosis II model, the inhibition of trans-signaling did not mitigate bone loss, highlighting the specificity of disease biology (140).

#### 4.4.5 Blocking IL-6 signaling *in vivo* (therapeutic angle)

In adolescent mouse models of ischemic osteonecrosis/bone injury, IL-6 receptor blockade induced early osteoclast recruitment, improved callus organization, and enhanced healing metrics (45), providing evidence that dampening excessive IL-6 can normalize bone repair dynamics *in vivo*.

## 5 Clinical and subclinical evidence: IL-6 and bone health

In adult and elderly populations, low-grade systemic inflammation and cytokine release driven by disease activity are consistently associated with decreased BMD, increased bone loss, and elevated fracture risk, with IL-6 and sIL-6R identified as significant correlates in both population and disease cohorts (27, 141).

### 5.1 IL-6 in pediatric inflammatory conditions and bone health:

In the context of juvenile inflammatory illnesses, it is demonstrated that elevated levels of inflammatory cytokines, particularly IL-6, are associated with developmental retardation and impaired bone health. In pediatric patients with JIA, increased osteoclastogenesis, decreased osteoblastic activity, disrupted ossification, and a diminished mineral apposition rate are correlated with *in vivo* over-expression of IL-6 (129).

Hence, IL-6 is found as a gene/signal pathway and endocrine factor associated with reduced BMD in children with idiopathic scoliosis (142).

Moreover, while direct studies on IL-6 and growth plate cartilage in pediatric cohorts are limited, prior mechanistic findings have recognized IL-6 as a cytokine that may directly affect growth plate cartilage, facilitating apoptosis and obstructing bone formation during chronic inflammation (143).

These findings suggest that IL-6 has been discussed in pediatric settings of chronic inflammation (JIA, IBD, scoliosis) with secondary bone/growth issues but direct clinical studies of IL-6 levels vs longitudinal growth or bone formation in healthy children remain sparse.

### 5.2 IL-6-induced inflammatory rheumatic disease (RA/JIA) and bone outcomes

Generalized bone loss is caused by Rheumatoid arthritis (RA) through inflammatory pathway, such as IL-6. IL-6 promotes RANKL expression, which leads to bone destruction by activating osteoclasts (144). Although adult patients with various arthritis have high IL-6 concentrations in their serum and synovial fluid, joint-resident cells (chondrocytes, synoviocytes, fibroblasts, and endothelial cells) lack the mIL-6R (145). Thus, IL-6 does not affect these cells. Complexing IL-6 with its soluble receptor may be the main mechanism by which it affects arthritis. Increased sIL-6Rα levels in synovial fluid from arthritic patients are associated with joint degeneration and the progression of RA. *In vitro* studies highlights a pivotal role of sIL-6Rα in synovial proliferation and expansion, bone resorption, and other inflammatory processes (146). TCZ may help in protecting bones in RA by potentiating BMD or causing a slight rise in it (147). In pediatric JIA, use of TCZ like biologic agents have similarly demonstrated improvements in growth trajectory and bone accrual (91, 148). However, it is observed that biologics, like IL-6 pathway inhibitors, are associated with advantageous outcomes in the stabilization or improvement of BMD and bone turnover markers (BTMs) in patients suffering from RA (149).

Use of biologics in JIA also demonstrated that TCZ is an effective drug for maintaining the bone accrual (150). Therefore, this emphasizes the clinical rationale for addressing IL-6–mediated inflammation in pediatric patients, despite the dearth of direct BMD trials.

### 5.3 Interventional human studies: IL-6 blockade and bone markers

In healthy adult and adult human with obesity, a regulated human physiology study (three placebo-controlled trials) demonstrated that acute IL-6R inhibition TCZ or exogenous IL-6 did not modify serum C-terminal telopeptide of type I collagen (CTX) or Procollagen type 1 N-terminal propeptide (P1NP) responses to exercise or mixed meals in the short term, indicating that acute IL-6 signaling may not directly influence circulating bone turnover markers in healthy or individuals with obesity (151).

In RA cohorts characterized by chronic inflammation, observational and longitudinal data indicate stabilization of BMD and advantageous shifts in bone turnover markers (BTM) with TCZ over several months. This suggests bone protection through the reduction of disease activity rather than a uniform acute endocrine effect on bone (147, 152).

Recent studies indicate that individuals with diminished femoral BMD may experience a comparatively bigger skeletal benefit from IL-6 inhibitors than from TNF inhibitors, suggesting BMD as a viable stratification tool in rheumatoid arthritis management; this theory is under investigation and requires prospective validation (153).

In both general populations and individuals with chronic inflammatory disorders, heightened IL-6 signaling, particularly through its soluble receptors is significantly correlated with reduced BMD, accelerated bone loss, and high risk of fracture. Elevated IL-6 levels enhance the osteoclast activity and halts the osteoblast function; thus, it promotes skeletal fragility. In contrast to it, however, pharmacological inhibition of IL-6, demonstrated through IL-6 receptor antagonists like TCZ, consistently maintains the BMD and normalizes bone turnover indicators, especially RA. Therefore, these effects mostly arise from low inflammation rather than a direct endocrine impact of IL-6 on bone (27, 147).

In short, the clinical and translational evidence highlights IL-6 as a critical connection between bone health and inflammation. Although, little IL-6 activity may expedite tissue regeneration, its sustained rise undermines bone formation and structural integrity. Elucidating this functional dichotomy will be essential for formulating medicines that reconstitute the skeletal equilibrium without encountering the normal immune function.

## 6 Stress-induced IL-6 and bone-related pathological conditions

**Figure 3:**
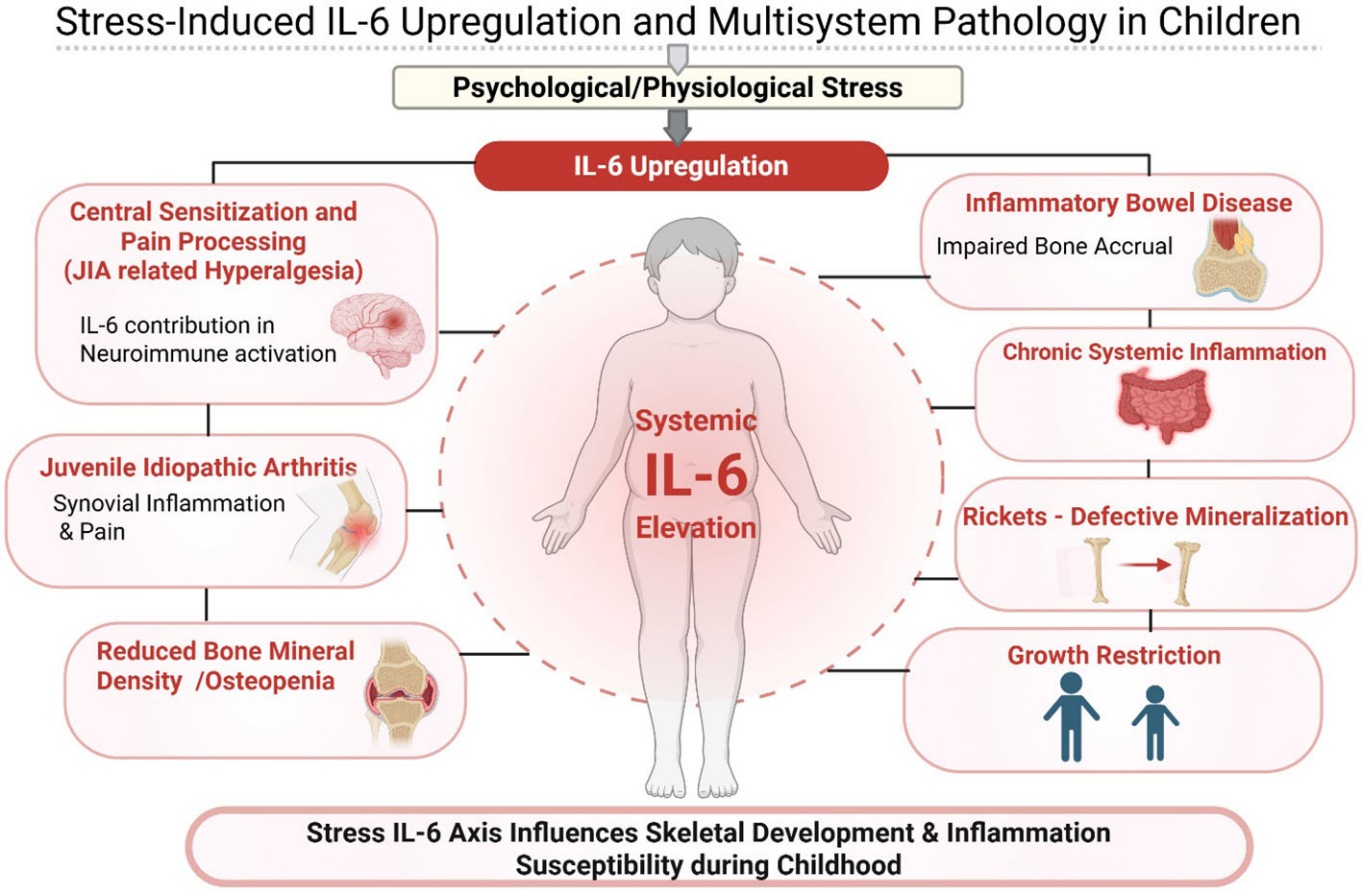
Stress-induced IL-6 upregulation and multisystem pathology in children. Stressors stimulate IL-6 upregulation, that leads to prolonged systemic IL-6 elevation in childhood. Elevated IL-6 then acts as a central inflammatory mediator bridging stress biology to multisystem consequences. In the musculoskeletal system, chronic IL-6 contributes to diminished bone mineral density (osteopenia), defective mineralization (rickets), impaired bone accrual in IBD (inflammatory bowel disease), juvenile idiopathic arthritis–associated synovial inflammation, and overall halt the growth leading to growth restriction. Beyond skeletal effects, IL-6 also participates in the central sensitization and pain processing such as JIA-related hyperalgesia in which it promotes chronic systemic inflammation. The figure illustrates how the stress–IL-6 axis bridges neuroimmune activation with skeletal development and inflammation during childhood.

As discussed earlier, neuroendocrine pathways (sympathetic–adrenomedullary and HPA-axis signaling) are activated by psychological stress that can shift immune activity toward a pro-inflammatory profile, producing cytokines such as IL-6, a measurable biomarker of stress-related immune activation (5). Chronic inflammatory states with elevated levels of inflammatory cytokines, particularly in children, are known to adversely affect bone health and impair bone mass by disturbing the balance between bone resorption and bone formation (154, 155). In adult humans, acute laboratory stressors reliably produce a post-stress increase in circulating IL-6, supporting a causal ‘stress to IL-6 rise’ relationship under controlled conditions (13). Higher levels of inflammatory biomarkers in later life are associated with exposure to psychosocial stress during childhood/adolescence, and IL-6 is redundantly included among these inflammatory outcomes (5). Furthermore, experimental paradigms showed that salivary IL-6 can be raised by acute psychosocial stress, demonstrating that IL-6 responses to stress are detectable in non-invasive matrices commonly used in pediatric settings (156). In pediatric inflammatory disease, specifically in children with polyarticular juvenile rheumatoid arthritis (a term that overlaps with the current JIA categories), leukocytes from these patients showed catecholamine-linked IL-6 induction through adrenergic mechanisms, suggesting that stress-system signaling can couple to IL-6 production in disease contexts (157). IL-6 production was increased by experimentally induced stress via leukocytes in children with juvenile rheumatoid arthritis, which indicates stress may magnify IL-6 responses, particularly in inflammatory phenotypes (158). In short, stress can upregulate IL-6 in humans, and pediatric inflammatory conditions may show heightened stress-immune coupling (**Figure 3**), while the consistency and magnitude of this effect differ by context and population (5, 13, 158).

### 6.1 Stress-induced IL-6 regulation and joint pain

IL-6 is a pleiotropic cytokine that rises rapidly during infection or tissue injury but becomes pathogenic when sustained, contributing to chronic inflammation and autoimmunity (159). Juvenile idiopathic arthritis (JIA) is a prime pediatric example in which IL-6 is fundamentally involved with systemic features, synovial inflammation, and downstream tissue effects, thus making IL-6 biologically and clinically relevant to joint pain and disability (160). Variations in IL-6 and associated molecules can not only influence disease manifestations but also significantly affect therapy outcomes in JIA (160, 161). Randomized trial evidence of systemic JIA explained that IL-6 receptor inhibition with TCZ results in substantial clinical improvement, thus confirming a causal relationship between IL-6 signaling and both systemic and articular disease activity (22). Joint pain arises in inflammatory arthritis occur from peripheral inflammation and modified pain processing, making IL-6 important for both synovitis and pain sensitization pathways. In a JIA model, persistent rise in spinal IL-6 associated with central sensitization and prolonged hyperpathia; halting the spinal IL-6 activity diminished pain behaviors and restored neural function (162). In JIA, high IL-6 levels have been linked to poor developmental outcomes, that includes reduced growth velocity, suggesting that IL-6 may represent and possibly modulate the inflammatory burden with musculoskeletal implications (163).

In short, stress can increase IL-6 levels, which may exacerbate joint pain through inflammatory amplification, including trans-signaling and pain sensitization. The most robust pediatric evidence pertains to JIA/systemic inflammatory arthritis rather than nonspecific musculoskeletal pain.

### 6.2 Stress-induced IL-6 regulation and bone deformities in children

Acute psychological stress frameworks in adult humans consistently show a rise in circulating IL-6 levels following stress stimuli, thus supporting the hypothesis that “stress leads to IL-6 upregulation” in a regulated environment (13). Pediatric-specific stress-IL-6 data are available from adolescent cohort and JIA studies (158). Consistently high levels of IL-6, trait of chronic inflammation, can impact skeletal growth through systemic hormonal activity and localized effects on the growth plate (164–166). In studies of Kashin-Beck Disease (KBD), an endemic bone and cartilage disorder mainly seen in children in certain regions, increased oxidative stress and ROS have been consistently observed (167). Rat models and human KBD tissue further demonstrate increased chondrocyte apoptosis alongside altered cytokine environments reinforcing the mechanistic paradigm in which oxidative stress promotes cartilage damage (168).

### 6.3 Stress-induced IL-6 regulation and rickets

Rickets is characterized by skeletal abnormalities such as bowed legs, enlarged wrists, and rachitic rosary, caused by poor mineralization during development (169). Besides deficiencies of vitamin D (1, 25-dihydroxyvitamin D) and calcium, inflammatory cytokines such as IL-6 have been shown to influence bone turnover and mineralization (103). IL-6 acts as a disease-modifying mediator in rickets rather than a primary cause, and its pathological relevance is strongest in inflammation-associated and Fibroblast Growth Factor 23 (FGF23)-driven rickets, where IL-6 links chronic inflammation to hypophosphatemia, disturbed bone remodeling, and growth-plate dysfunction (114, 170–172). Vitamin D deficiency is a major cause of nutritional rickets and has been associated with increased oxidative stress (173). *In vitro*, active vitamin D (1, 25-dihydroxyvitamin D3) suppresses IL-6 production in human lymphocytes, suggesting that vitamin D deficiency may permit higher IL-6 levels (174).

Overall, this creates a vicious cycle in which Vitamin D deficiency increases the levels of ROS and IL-6, and this, in turn, worsens mineralization defects and inflammatory bone damage.

### 6.4 Stress-induced IL-6 and juvenile idiopathic arthritis

Children suffering from JIA present high oxidative stress which accompanies activation of chronic inflammatory cascade including IL-6 signaling (125, 126, 175, 176). IL-6 promotes osteoclast activity while it halts osteoblast activity, and overall, with ROS-driven remodeling imbalance, it results in low BMD (osteopenia) and enhances the risk of bone fragility during the growth-accelerative period (29, 129, 166). In patients with Juvenile Rheumatoid Arthritis (JRA), any type of stressor, whether emotional or physical stress, leads to significantly higher production of the inflammatory protein including IL-6 by leucocytes. This cascade response is not observed in healthy individuals and mediated by the presence of α1-adrenergic receptors present on the immune cells of JRA patients, thus creating a direct pathway between stressors and increased inflammation (175).

### 6.5 Stress-induced IL-6 regulation and IBD

Inflammatory bowel disease, often known as IBD is a collective name for Ulcerative Colitis and Crohn’s disease, where both are main types of gut inflammation and both present almost similar symptoms but affect different regions of GI tract (Crohn’s disease: can cause inflammation anywhere from the mouth to anus, Ulcerative Colitis: only affects large intestine) (177, 178). In mouse models, psychological stress such as repeated water-avoidance stress, prenatal stress or social disruption can increase IL-6 levels in intestinal tissue and/or circulation and mediate colitis severity (179–181). In pediatric IBD, inflammation is found to be a detrimental factor for bone loss in the chronic disease state, correlating with high serum IL-6 levels affecting bone density (182, 183). In short, mechanistically, OS and IL-6 amplify osteoclast activity and impair accrual that leads to low BMD and compromised skeletal growth in IBD-affected children, the mechanistic link between oxidative stress, IL-6, and bone loss is further supported by adult osteoporosis literature (182–184).

### 6.6 Stress-induced IL-6 regulation and pediatric systemic lupus erythematosus

Systemic lupus erythematosus (SLE) is an autoimmune disease representing a clinically heterogeneous spectrum of symptoms and problems with a relapsing and remitting course (185). OS has a pivotal role in this process; normally, Treg cells help in controlling the immune system as well as preventing it from attacking the body. In SLE patients, both the number and function of Treg cells are found to be lowered. Oxidative stress causes an increase in IL-6 levels, which suppresses a key protein known as Foxp3, which is needed for Treg cell development. As a result, fewer Treg cells are produced, leading to an immune imbalance in SLE (186–190). Bone involvement is one of the major and devastating features of SLE including issues like Osteomyelitis (OM), Osteoporosis (OP) and Avascular necrosis (AVN), and these bone-related issues are more frequent in SLE patients as compared to the normal population. In adult SLE patients, oxidative stress is linked to disease activity; cytokine profiling in childhood-onset SLE (pSLE) similarly reveals elevated inflammatory mediators including IL-6, suggesting that redox imbalance and cytokine activation collectively contribute to disease pathophysiology in children (191, 192). ROS and IL-6 mediated osteoclastogenesis aggravate osteopenia and contribute to the disruption of peak mass acquisition as demonstrated by studies done in children and adults (29, 193–197).

### 6.7 Stress-induced IL-6 dysregulation and kwashiorkor

Kwashiorkor is a protein-energy malnutrition (PEM) found in children with extremely reduced dietary patterns (famine) (198). Different inflammatory molecules are studied in Kwashiorkor including IL-6 and antioxidant profiling, showing reduced levels of antioxidant molecules, depicted increment in oxidative stress in children with protein-energy malnutrition (198, 199). PEM leads to growth retardation, poor weight gain, and delay in developmental goals in children (200). Thus, in short, IL-6 and oxidative stress collectively worsen the consequences of PEM and cause multiple health complications including skeletal muscle loss, disturbance in circulating amino acid quality and quantity, thus leading to severe growth retardation (198, 199, 201).

## 7 IL-6 treatment strategies

The drugs which are used in inflammatory bone-related issues as an approved clinical treatment are mentioned in **Table 2**, which includes TCZ (ACTEMRA^®^; anti-IL-6R): FDA-approved for systemic JIA (≥2 y) and polyarticular JIA (≥2 y); also for cytokine-release syndrome (CRS) (including pediatric) (202, 203). Sarilumab (KEVZARA^®^; anti-IL-6R): In June 2024, the FDA approved Sarilumab for polyarticular JIA in patients ≥63 kg; adult approvals include RA and others (206–208). Labels focus on disease control; bone benefits in children are secondary to reducing inflammatory activity (e.g., improved growth/BMD observed when disease control is achieved). Formal pediatric BMD endpoints are still scarce.

**Table 2:** Summary of approved and commonly used therapeutic agents targeting IL-6 or inflammatory pathways in pediatric inflammatory bone-related conditions.

Drug name	Type/target	Approved indications	Pediatric use	Bone-related benefits	Notable risks/side effects	References
TCZ (ACTEMRA^®^)	Anti-IL-6R monoclonal antibody	Systemic JIA (≥2 yrs) - Polyarticular JIA (≥2 yrs) - Cytokine Release Syndrome (CRS, incl. pediatric)	FDA-approved for ≥2 years	low IL-6 leads to low inflammation leading to improved BMD, growth, less skeletal loss	Infections, neutropenia, increased liver enzymes, increased lipids	(21, 22, 159, 202–205)
Sarilumab (KEVZARA^®^)	Anti-IL-6R monoclonal antibody	RA (adults) - Polyarticular JIA in children ≥63 kg (FDA approved June 2024)	Approved for ≥63 kg (2–17 yrs studied)	Reduces inflammation giving indirect bone benefit	Neutropenia; requires careful monitoring	(206–209)
Siltuximab	IL-6-neutralizing monoclonal antibody	Multicentric Castleman Disease with skeletal involvement	Rare pediatric use under specialist supervision	reduces IL-6 signaling thus improving osteopenia, bone pain	Use in children limited; specialist only	(44, 210, 211)
Prednisolone (Systemic Glucocorticoid)	Anti-inflammator; indirect IL-6 suppression	Inflammatory/rheumatic diseases	Widely used in pediatric rheumatology	Short-term use reduces inflammation; long-term use causes bone loss	low BMD, high fracture risk, growth suppression, endocrine toxicity	(44, 211–216)
Methotrexate & Conventional DMARDs	Immunomodulator; indirect IL-6 reduction	JIA, RA, other inflammatory diseases	Commonly used in pediatric JIA; often with biologics	Lowers overall inflammation, gradual reduction in IL-6 and bone resorption	Liver toxicity, cytopenias, GI symptoms	(217)

This table highlights IL-6–targeted biologics and conventional anti-inflammatory therapies that are used in the clinical management of conditions with bone involvement.

IL-6 signaling pathway - targeted biologics are the most direct clinical option when role of IL-6 is assured as a key player for inflammatory disease activity (e.g., systemic juvenile idiopathic arthritis (sJIA), cytokine release syndrome (204).

**Figure 4:**
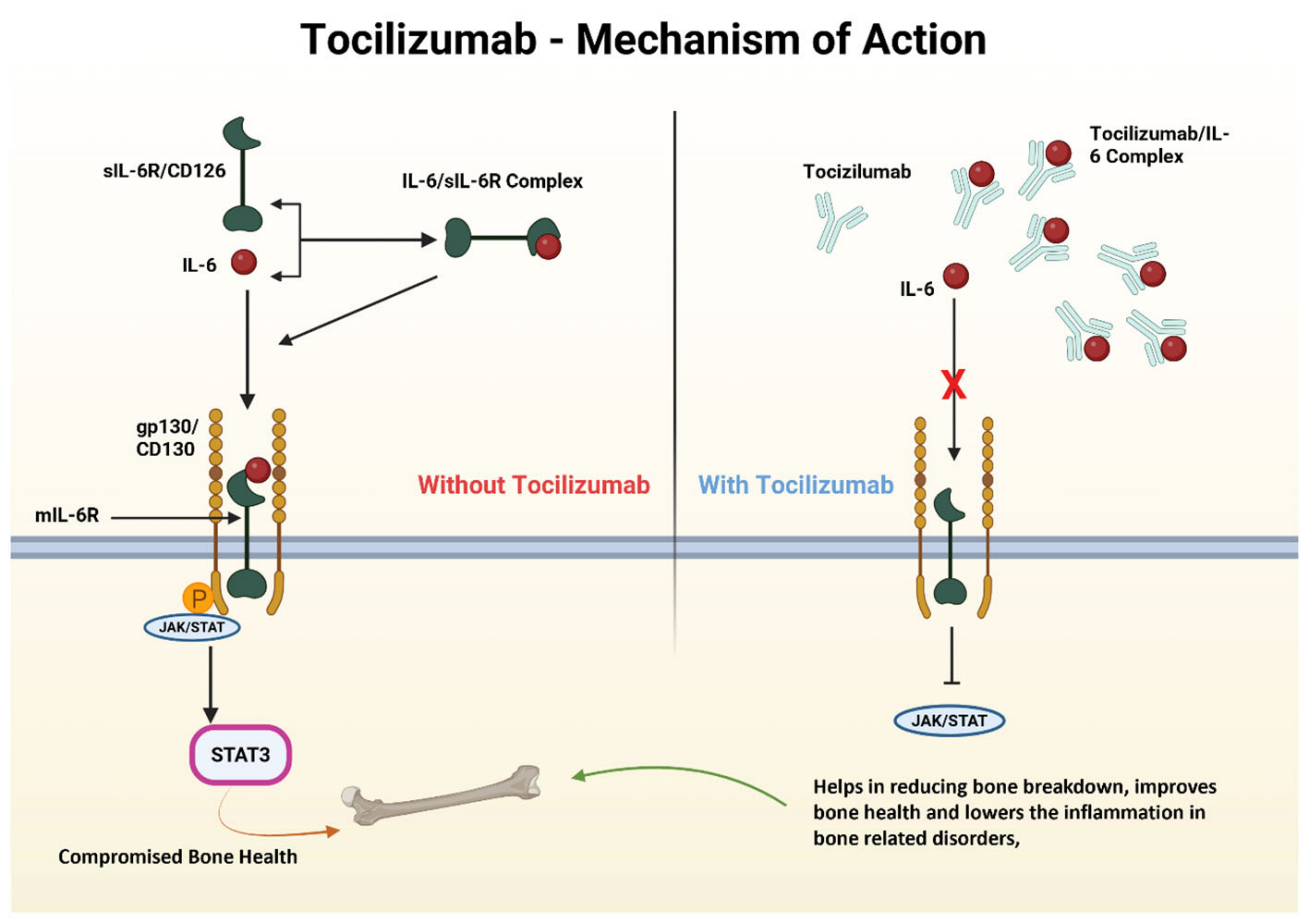
Mechanism of action of TCZ blocking of IL-6 binding with IL-6R and blockage of downstream signaling cascade lowers inflammation, bone pains and swelling.

TCZ is a humanized monoclonal antibody designed to bind both mIL-6R and sIL-6R and inhibits downstream signaling, leading to reduced CRP and overall inflammation (22). In JIA, IL-6 acts as a central pathogenic cytokine that drives along synovial inflammation, systemic issues, and growth failures (21). TCZ is an IL-6 blocker that halts the JAK/STAT signaling pathway, reduces cytokine-associated osteoclastogenesis, improves the inflammatory profile, and thus indirectly helps bone by reducing inflammation-mediated resorption (22) as shown in **Figure 4**. Long term follow ups showed improved growth velocity and less skeletal loss in treated children (218). The main practical risks with TCZ are serious infections, cytopenias such as neutropenia, liver enzyme elevations and elevated lipid levels (205).

**Figure 5:**
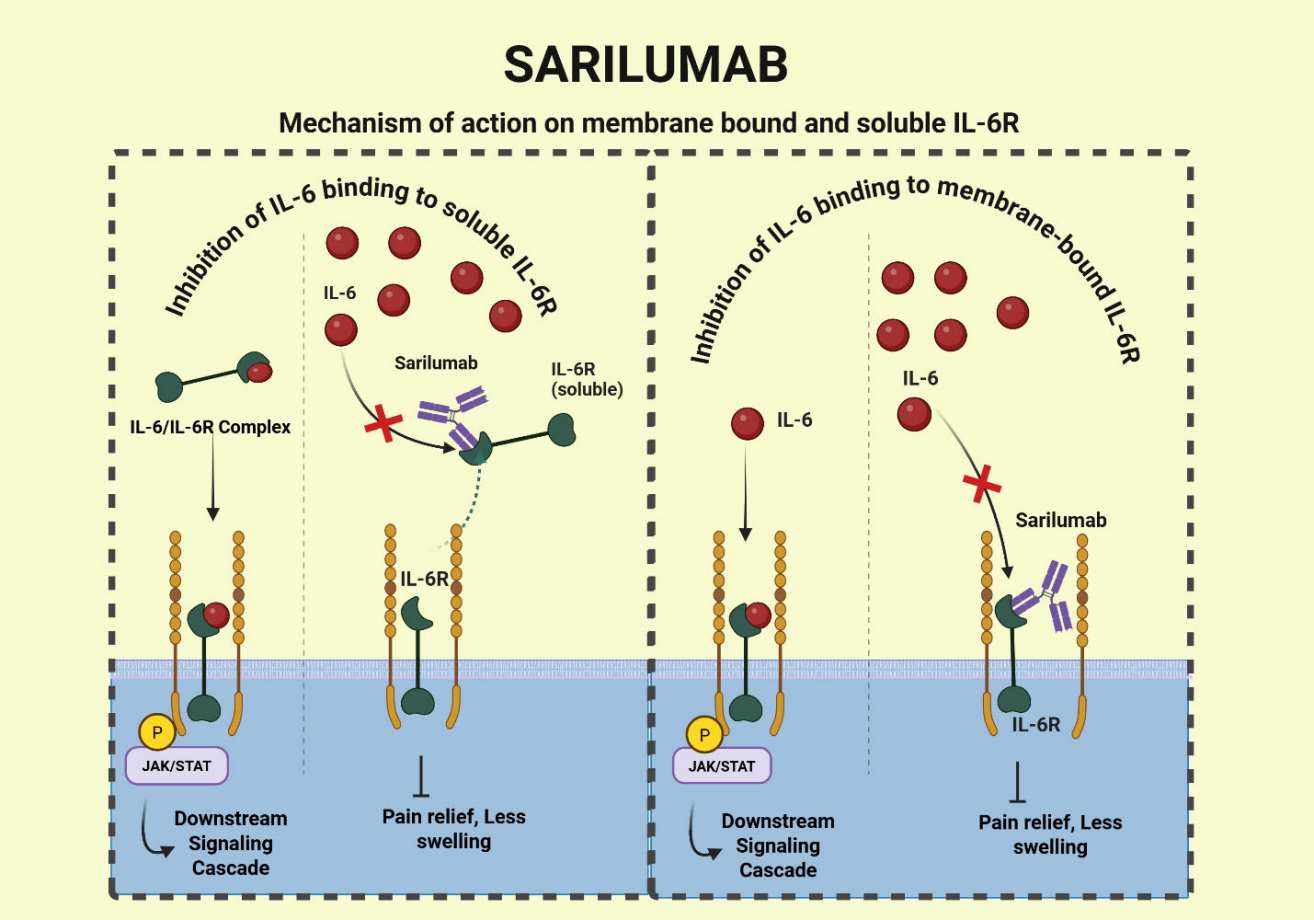
Mechanism of action of Sarilumab, which binds to IL-6R and blocks binding to ligand IL-6 molecules inhibiting downstream signaling cascade and lowering the pain and inflammation in patients.

Sarilumab exhibited efficacy for treating RA in adult patients but has shown promising results in patients of Polyarticular-Course Juvenile Idiopathic Arthritis from 2 years to 17 years of age as well, with almost the same dosage as recommended for adults, but under specialist guidance, as neutropenia could be a side effect of the treatment and needs proper monitoring (219). Mechanism of action is illustrated in **Figure 5**.

In multicentric Castleman disease with skeletal involvement, IL-6 levels are found to be high, causing inflammation in the body, and osteopenia with bone pains (209, 220). Siltuximab is a drug used to reduce systemic inflammation by neutralizing the circulating IL-6 in the body, as well as improve bone-related manifestations by reducing the downstream IL-6 signaling (210) however, its use in pediatric populations remains rare, as safety has not been established, and it is generally administered only under strict specialist supervision.

Several commonly used anti-inflammatory drugs can indirectly reduce IL-6 by suppressing upstream immune activation rather than targeting IL-6 specifically (44). Systemic exogenous GCs can rapidly lower inflammatory cytokine production and symptoms; however, high doses and long-term use of GCs may cause skeletal and endocrine side effects, including decreased bone growth and BMD as well as increased rates of vertebral/morphometric fractures (211–213). In GC-induced osteoporosis secondary to inflammatory rheumatic diseases, bone is indirectly protected by IL-6 targeted biologics by minimizing long term steroid exposure (214, 215). When GCs are used at high doses for long-term treatment, although they suppress inflammatory cytokines, they may also trigger undesired side effects in the form of apoptosis, reduced proliferation and differentiation. To reduce GC-induced adverse effects, triblock copolymer, based systems have also been developed to enable controlled GC release under specific conditions; however, these drug delivery systems have not yet been explored *in vivo* (221). Mechanistically, lowering IL-6 activity limits direct suppression of bone formation and growth plate toxicity, while lowering steroids inhibits inflammatory bone resorption and permits osteoblast function to recover (211, 216). Methotrexate and other conventional Disease-Modifying Antirheumatic Drugs (DMARDs) have shown to reduce the level of IL-6 gradually in cases of adult inflammatory arthritides as overall inflammatory burden is controlled in the body, and they are usually co-prescribed with biologics in clinical rheumatology practices (217). In a pediatric study of juvenile rheumatoid arthritis, methotrexate significantly lowered IL-6 production from activated immune cells, supporting its role in modulating inflammatory cytokines (222)

## 8 Stress, IL-6 and anorexia

In the presence of psychological or physiological stress, the activation of neuroendocrine stress pathways stimulates IL-6 production, which, in turn, exacerbates anorexia by fostering inflammation-induced illness behavior and inhibiting central appetite-regulating circuits (130, 223, 224). It can contribute to anorexia, particularly in chronic inflammation and sickness behavior, though it is often part of a broader cytokine-mediated network rather than acting in isolation (225, 226). Central and parabrachial IL-6 administration in animal models decreases body weight and food intake, suggesting a direct impact on the brainstem and hypothalamic signaling pathways that control appetite and fullness (227). Elevated circulating IL-6 has also been correlated with decreased appetite in humans after high-intensity exercise, reflecting transient sickness-like behavioral responses (228). The mechanisms involve interactions with other cytokines, including IL-1β, and the modification of hypothalamic neuropeptides that inhibit eating during inflammation (224). Thus, stress-induced IL-6 represents a mechanistic link between psychosocial stress and anorexia rather than a nonspecific inflammatory association.

## 9 IL-6 and body mass index

A clear association between high IL-6 levels and low BMI has also been reported, suggesting the presence of chronic inflammatory disease such as cachexia (a syndrome characterized by involuntary loss of weight, metabolic dysregulation and skeletal muscle wasting) rather than obesity-related inflammation (229, 230). High IL-6 levels can also result in high BMI, weight loss, or chronic inflammation in lean individuals.

High levels of circulating IL-6 are a potential mediator of inflammatory cachexia observed in chronic diseases like cancer, rheumatoid arthritis, and chronic systemic infections (231). IL-6 triggers muscle proteolysis through JAK/STAT3 pathway activation and enhances the production of ubiquitin–proteasome components, while it enhances lipolysis in adipose tissue simultaneously, thereby contributing to loss of both lean and fat mass (232, 233). Upregulation of IL-6 causes body weight reduction, which is independent of caloric intake, while IL-6 blockade attenuates muscle wasting in tumor-bearing models, demonstrating its causal role in catabolic states (230, 233). IL-6 signaling inhibition in Rheumatoid arthritis patients caused a potential increase in lean body mass of the patients and also aided improvements in overall body composition, thus demonstrating reversal of inflammation-induced muscle wasting (234). Furthermore, IL-6 blockade altered metabolic parameters, such as lipid profiles, manifesting that IL-6 has a central role in maintaining both body composition and systemic metabolic balance in chronic inflammatory conditions. Similarly, a comparative study comprising of 16 weeks showed that patients who received pharmacological IL-6 blockade experienced more weight gain than those patients who were treated with TNF-α inhibitors. This is an important experimental work suggesting a prominent role of IL-6 inhibition in exponentiating the body weight during the treatment regimen of inflammatory rheumatic disorders (235).

However, IL-6 conversely contributes to the pathophysiology of obesity and metabolic syndrome as well. In adiposity-induced inflammation, hypertrophic adipocytes and infiltrating macrophages cause a rise in IL-6 secretion, which promotes insulin resistance and triggers hepatic CRP production (236, 237). It is interesting to know that IL-6-deficient mice developed mature-onset obesity, depicting the importance of basal IL-6 signaling, which is involved in energy balance and lipid metabolism regulation (238). In murine models, central delivery of the IL-6 gene into the brains of rats upregulated uncoupling protein-1 (UCP1) expression in BAT and decreased body weight, demonstrating that brain-targeted IL-6 can trigger thermogenic pathways (239). However, this effect was diminished when sympathetic innervation to BAT was blocked, ensuring that IL-6-triggered weight loss relies on the activation of the sympathetic nervous system of brown fat thermogenesis, underscoring the complex and context-dependent metabolic role of IL-6.

Elevated levels of IL-6 are also observed in lean/thin individuals with chronic inflammatory conditions or stress-related issues, and this indicates that systemic inflammation can occur independently of BMI (240, 241). In such contrasting cases, IL-6 represents immune activation rather than adiposity per se. In lean individuals, persistent low-grade elevation of IL-6 has been correlated with high cardio-metabolic risk and features of “inflammaging, “ further highlighting that BMI alone may not factually capture inflammatory burden (241, 242). Astrocyte-specific overproduction of IL-6 in transgenic mice helped them stay protected against high-fat diet-induced weight gain and fat accumulation (243). This finding indicates that central nervous system-derived IL-6 increases energy expenditure and promotes resistance to diet-induced obesity, therefore highlighting a neuro-metabolic regulatory role of IL-6. IL-6 regulates potential hypothalamic neuropeptides, which are involved in appetite-energy balance regulation. IL-6 modulates neuropeptide expression, such as neuropeptide Y (NPY) and proopiomelanocortin (POMC), thereby demonstrating a direct involvement in the central control of food intake and body weight (244). These regulatory effects were gender-dependent, proposing sex-specific dissimilarities in IL-6-mediated neuroendocrine control of metabolism.

Altogether, these data highlight a bidirectional and context-specific association between IL-6 and BMI. While obesity plays a role in elevating IL-6, persistently increased IL-6 levels can themselves trigger metabolic alterations, muscle-related catabolism, or dysregulated adiposity. Thus, interpretation of IL-6 levels should consider both nutritional status and underlying inflammatory problems, especially in pediatric populations where growth, body composition, and maturation of the immune system intersect.

## 10 Strategies to inhibit IL-6 signaling

The latter pre-clinical interventions to inhibit IL-6 signaling s do not focus on complete cytokine ablation, reflecting concerns regarding growth and immune maturation; rather, they focus on pathway-selective, multi-target, and modality-expanded approaches (245) as shown in **Table 3**. One of the leading strategies is to inhibit IL-6 trans-signaling partially using sgp130Fc/olamkicept (a decoy receptor fusion protein) that binds ligand/receptor (IL-6/sIL-6R) complex and averts its interaction with membrane gp130, thus, pro-inflammatory trans arm is being blocked and IL-6 molecule is spared for classical signaling without causing immune suppression; target-engagement and efficacy have been studied in models like IBD and SARS-Cov2 mice models (248, 252, 253).

**Table 3:** Emerging pre-clinical therapeutic strategies targeting IL-6 signaling pathways.

Strategy/modality	Target & mechanism	Experimental model	Key findings/rationale	Age group	References
Anti-IL-6R monoclonal antibody (TCZ) in juvenile ischemic osteonecrosis (JIO)	IL-6 receptor blockade (TCZ, humanized anti-IL-6R mAb); inhibits IL-6/sIL-6R-driven cartilage destruction and osteoclastogenesis in the developing femoral head	Immature mouse model (~6-week-old mice) with ischemic osteonecrosis	TCZ reduced chondrocyte apoptosis, increased cartilage matrix and significantly increased bone volume and thickness	6-week-old (juvenile) mouse model of JIO; treated for 6 weeks	(246)
Anti-IL-6R antibody (MR16-1) in adolescent JIO (juvenile ischemic osteonecrosis)	IL-6 receptor blockade using MR16-1 (murine-specific anti-IL-6R); reduces RANKL-dependent osteoclastogenesis and promotes osteoblast-driven bone repair in ischemic necrosis of the growing femoral head	Adolescent mouse model with ischemic osteonecrosis	IL-6R blockade significantly increased bone volume (Cohen’s d=2.0), trabecular thickness (d=2.3), osteoblast number (d=2.3), and bone formation rate (d=4.3). Increased revascularisation and restoration of necrotic marrow.	12-week-old skeletally immature adolescent mice were surgically induced with JIO	(49)
Anti-IL-6R monoclonal antibody (15A7) to block IL-6 signaling in transgenic mice	The antibody neutralized the murine IL-6 receptor, preventing IL-6 from binding and activating downstream gp130-STAT3 signaling. This rescued growth impairment by restoring IGF-I production, which IL-6 normally suppresses.	Transgenic mice overexpressing human IL-6 (NSE/hIL-6). Human validation included children with systemic juvenile rheumatoid arthritis (s-JRA).	IL-6 transgenic mice showed growth impairment (50-70% of normal size) due to reduced IGF-I despite normal growth hormone. Blocking IL-6R partially corrected growth defects. In s-JRA patients, higher IL-6 correlated with lower IGF-I, confirming translational relevance.	Transgenic mice were studied from early life. Human cohort: children (mean age 6.5 yr, range 2–17 yr) with systemic juvenile rheumatoid arthritis.	(51)
Combined Fc-OPG + hPTH sequential therapy in IL-6-overexpressing growing mice	Sequential antiresorptive (Fc-OPG, RANKL inhibitor) followed by anabolic (hPTH, parathyroid hormone) therapy to address both increased bone resorption and impaired bone formation caused by IL-6 overexpression in the growing skeleton	Preclinical juvenile bone disease model. Growing IL-6-transgenic (TG) mice with generalized bone loss, osteopenia, and stunted growth	Sequential Fc-OPG/hPTH treatment improved skeletal growth and prevented bone loss in growing IL-6 TG mice. First experimental therapy shown to improve both bone quantity and quality in a preclinical model designed to mirror pediatric chronic inflammatory bone disease.	IL-6 transgenic mice (TG), treated during their juvenile growth phase (day 4–30 of life). Directly relevant to pediatric bone biology.	(247)
TCZ in systemic JIA (Juvenile Idiopathic Arthritis)	By neutralizing IL-6R, TCZ prevents IL-6 from activating downstream gp130-STAT3 signaling, reducing inflammation and its negative effects on growth and bone metabolism.	Phase III RCT n=112 children aged 2–17 years with systemic JIA; *post-hoc* analysis of 83 patients for growth and bone biomarkers over 2 years	Children with stunted growth at baseline experienced significant catch-up growth during TCZ treatment. IGF-1 levels normalized. Bone formation and resorption markers both increased significantly. Direct pediatric clinical evidence that IL-6 inhibition rescues growth and improves bone homeostasis in children.	Pediatric patients (aged 2-17)	(148)
Selective IL-6 trans-signaling inhibition (sgp130Fc/Olamkicept)	Decoy gp130 fusion protein binds IL-6/sIL-6R complex, selectively inhibiting IL-6 trans-signaling while sparing classic IL-6 signaling	Murine inflammatory models (including colitis/IBD-relevant models); human PK/PD and mechanistic studies	Reduces pathological inflammation without full immune suppression; preserves homeostatic IL-6 functions	16 adult patients (ages 21-66) with active ulcerative colitis (UC) or Crohn’s disease (CD).	(248)
Bispecific anti-TNF/anti-IL-6 nanobodies	Simultaneous neutralization of TNF-α and IL-6, reducing synovitis and osteoclastogenic signaling	Collagen-induced arthritis (CIA) mouse model	Greater reduction in arthritis severity than mono-target blockade; mechanistic relevance for inflammatory bone loss	Adult mice	(249)
Anti-IL-6 nanobodies (VHHs)	High-affinity single-domain antibodies against IL-6 with improved tissue penetration and stability	Synthetic/humanized nanobody libraries; *in-vitro* binding and stability assays	Demonstrated feasibility of compact, stable IL-6 inhibitors for next-generation biologics	Not applicable	(250)
Small-molecule IL-6 signaling inhibitors	Disruption of IL-6/IL-6R/gp130 complex or gp130-mediated downstream signalling via PPI modulation	Structure-based drug design, molecular docking, early *in-vitro* validation	Offers oral, lower-cost alternatives to biologics; early proof-of-concept compounds identified	Not applicable	(251)

This table summarizes novel IL-6–modulating approaches beyond conventional global IL-6 blockade, including pathway-selective inhibition (e.g., IL-6 trans-signaling blockade with sgp130Fc/olamkicept), multi-target biologics (bispecific cytokine inhibitors), nanobody-based modalities, and small-molecule inhibitors of IL-6/gp130 signaling. For each strategy, the primary molecular target, mechanism of action, experimental model or technique, and key translational findings are outlined, highlighting their potential relevance for inflammatory and bone-related disorders characterized by IL-6 dysregulation.

Another strategy is to design dual-pathway biologics that overcome cytokine redundancy issues including bispecific formats like IL-6R + IL-17A, which has been evaluated in inflamed mouse models of delayed type hypersensitivity (DTH); this combined therapy blockade showed inflammatory suppression more effectively than monotherapy, supporting pre-clinical rationale for multi-target IL-6 approaches (254). Pertinently, bispecific anti-TNF/IL6 nanobody constructs have shown effective responses in Collagen-induced arthritis (CIA) mouse models, with mechanistic logic that TNF/IL-6 pathway suppression simultaneously reduces synovitis and downstream osteoclastogenic signaling more effectively than single-axis blockade (249). Another approach is the designing of anti-IL-6 nanobodies that contain VHHs (variable antigen-binding domains) using synthetic or humanized nanobody libraries, where researchers have reported high-affinity IL-6 binders and substantiated their persistence as a foundation for future drug delivery optimization and therapeutic developments (250, 255).

Lastly, to address the affordability and route of administration of biologics, low molecular weight antagonists of IL-6 signaling that target IL-6/IL-6R/gp130 complex are being engineered using structure-guided medicinal chemistry, emphasizing structure-based design, molecular docking, and protein-protein interaction (PPI) principles, with definitive experimental frameworks for optimization (38, 251, 256).

## 11 Foods and dietary patterns regulating IL-6: implications for bone health

Most large-scale dietary intervention studies targeting inflammatory cytokines such as IL-6 have historically been conducted in adult populations. However, emerging pediatric evidence shows that diet also plays an important role in the regulation of inflammatory markers in children and adolescents. Systematic reviews and epidemiological analyses show that diet quality and inflammatory dietary indices are significantly associated with circulating inflammatory biomarkers, including IL-6, in pediatric populations (257–261). Population-based analyses using NHANES data further demonstrated that pro-inflammatory dietary patterns, assessed using the Dietary Inflammatory Index (DII), are associated with adverse metabolic and inflammatory profiles in children and adolescents aged 6–19 years (258, 259). Beyond pediatric observational data, broader nutritional research indicates that diets with excessive fruits, vegetables, unsaturated fats and whole grains are associated with lower systemic inflammation. For example, adherence to Mediterranean-style dietary patterns has been associated with reduced circulating IL-6 levels in male adults (262). In adult individuals, interventions in humans having metabolic syndrome with anti-inflammatory dietary strategies resulted in decreases in IL-6, IL-8 and TNF-α (263). Consumption of red meat and decreased consumption of nuts and whole grains are associated with elevated IL-6 levels (264, 265). In healthy adults, the highest quartile of red meat intake exhibited significantly higher IL-6 levels and IL-8 compared to those in the lowest quartile (264). Pediatric-specific dietary data on this association is more limited. Additionally, single-nutrient studies indicate that particular diets may affect IL-6 levels: a daily optimum vitamin C supplementation decreased IL-6 plasma concentration in diabetic adults (266).

**Figure 6:**
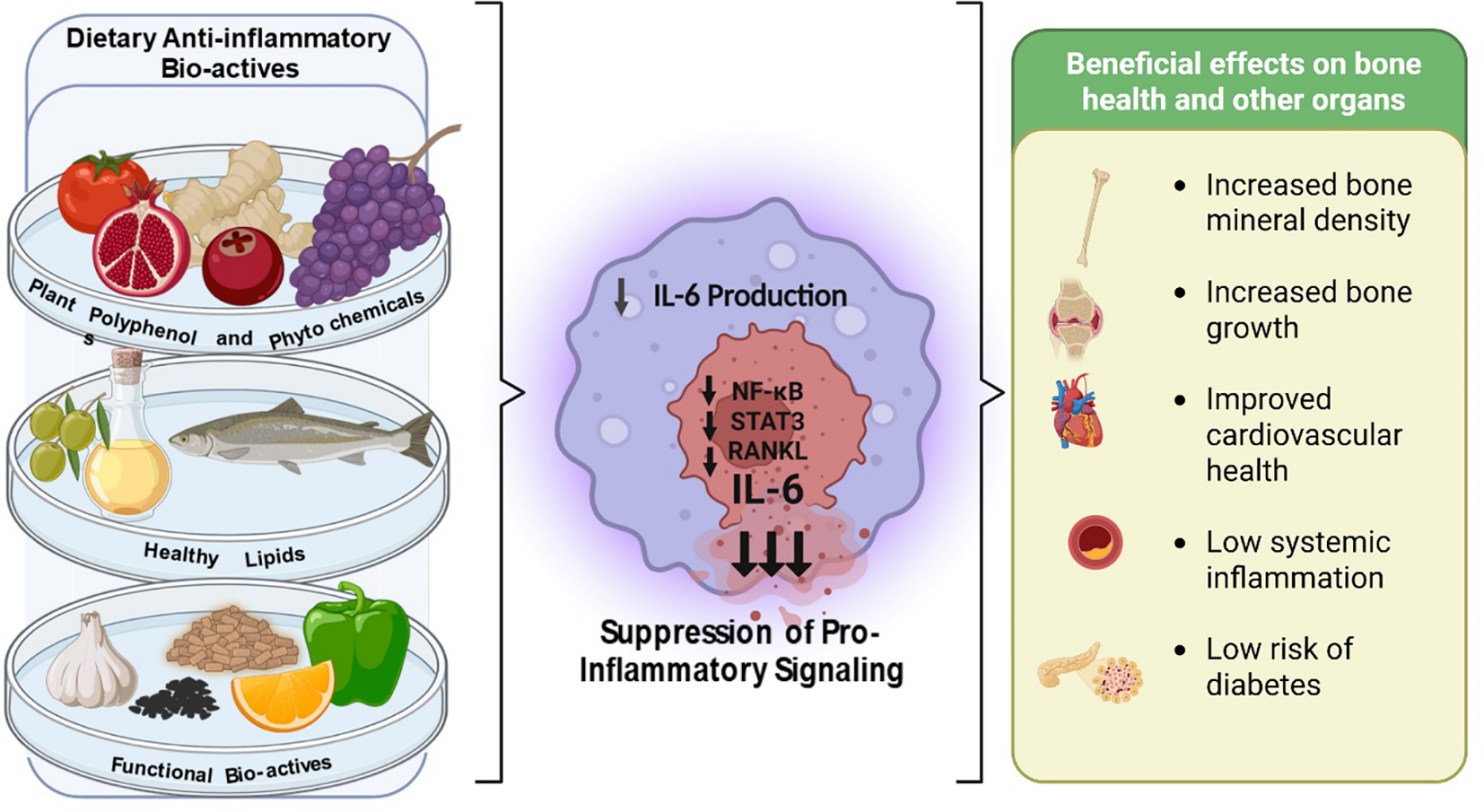
Dietary bioactives modulate IL-6 and inflammatory signaling. Polyphenols and phytochemicals, healthy lipids and other functional compounds can reduce IL-6 production by inhibiting key inflammatory signaling pathways such as NF-κB, STAT3, and RANKL. The suppression of IL-6 subsequently reduces osteoclast activity, supports bone health, and attenuates chronic systemic inflammation, thereby contributing to improved skeletal and cardiometabolic outcomes.

Although numerous dietary components have been investigated for their potential anti-inflammatory properties in adults, direct investigation of dietary modulation of IL-6 in pediatric populations remains limited. Current pediatric evidence primarily involves a small number of clinical studies evaluating nutritional interventions, often in the context of inflammatory conditions affecting bone or joint health. These studies suggest that certain dietary compounds may influence inflammatory pathways and bone-related outcomes in children and adolescents, although IL-6 is not always directly measured. A summary of dietary patterns and compounds reported to influence inflammatory biomarkers and bone health is provided in **Table 4**. **Figure 6** provides a graphical abstract summarized in **Table 4**, offering a clearer visual representation to facilitate understanding.

**Table 4:** Dietary and natural compounds with documented anti-IL-6 effects in human or animal studies.

Name of food/compound	Anti-IL-6 effect	Species tested	Target tissues studies	Pathological condition/disease	Age (juvenile/adult)	Reference
Lactoferrin (milk protein)	Yes	Humans (75 children)	Systemic immune cytokines (blood)	Failure-to-thrive with infection	Pediatric (1–5 yrs)	(267)
Probiotics (multi-strain)	Yes	Humans (100 children)	Oral mucosa/salivary cytokines	Gingival inflammation	Pediatric (~10 yrs)	(268)
Probiotics (VSL#3 – multi-strain)	Yes	Humans (Children, mean age 15 ± 2.5 yrs)	Joints/Systemic (serum cytokines)	Juvenile Idiopathic Arthritis – Enthesitis-Related Arthritis (JIA-ERA)	6–20 years (pediatric to young adult)	(269)
Sweet cherry polyphenols (Prunus avium)	Yes (indirect through reduction of TNFα)	Humans (25 children with obesity, *in vitro* PBMCs)	Bone/osteoclast activity	Childhood obesity-related osteopenia	Pediatric (mean 10.8 ± 2.6 yrs)	(270)
Curcumin/turmeric	Yes	Humans (pediatric, 16 trials)	Joints, immune/systemic	JIA, IBD, asthma, metabolic disorders	Pediatric	(271)
Pomegranate	Yes	Cell lines of mouse calvaria	MC3T3-E1 cells (RCB1126, an osteoblastlike cell line from C57BL/6 mouse calvaria	Inflammatory bone loss (Osteoblast dysfunction)	–	(272)
Nigella sativa Black seed	Yes	Rats	Bone	Osteoporosis	Adult	(273)
Berries (Blueberries, Strawberries)	Yes	Humans	Bone	Knee Osteoarthritis	Adult	(274)
Extra virgin olive oil	Yes	Mice	Bone	Arthritis	Adult	(275)
Vitamin C (Ascorbic acid)	Yes	Humans	Muscle	Exercise-induced muscle IL-6 release	Adult	(276)
Curcuma longa (Curcumin)	Yes	Humans	Systemic (circulating cytokines)	Metabolic Syndrome	Adult	(277)
Ginger (Zingiber officinale)	Yes	Humans	Low grade Chronic Inflammation in Blood via Systemic Plasma cytokines	type 2 diabetes (DM2),	Adult	(278)
Gralic (Allium sativum)	Yes		A Systematic Review and Meta-analysis of 16 Randomized Controlled Trials	Inflammatory proteins	Adult	(279)
Boswellia serrata extract	Yes	Human	Knee	degenerative hypertrophy OA	Adult	(280)
Capsicum annuum (Capsaicin)	Yes	Mice	Systemic inflammation (serum cytokines)	Obesity	Adult	(281)
Tomatoes/Lycopene	Yes	Rats	Adipose + systemic (plasma IL-6)	Obesity	Adult	(282)
Olive oil/Mediterranean diet with VOO	Yes	Humans	Heart	Atherosclerosis	Adult	(265)
Fatty fish/Omega-3 (fish oil)	Yes	Humans	Bone	active rheumatoid arthritis	Adult	(283)

Various foods, plant-derived compounds, and nutrients have been reported to exert suppressive effects on the expression of IL-6. Evidence from human and animal studies highlights the potential role of dietary interventions and phytochemicals in mitigating inflammation through the regulation of IL-6 across different tissues and organs affected by inflammatory conditions.

***Implications for bone health:*** Given that elevated IL-6 is mechanistically associated with increased bone resorption, impaired osteoblast activity, and reduced bone formation in both preclinical and clinical studies, dietary patterns that lower IL-6 levels may offer additional benefits for bone health, particularly in children, adolescents, and individuals with chronic inflammatory conditions. However, to date, no comprehensive pediatric bone health studies have directly investigated the dietary intake, IL-6, and bone growth as a cohesive study model.

## 12 Nutrient adequacy, stress and IL-6

Nutrient adequacy refers to meeting energy requirements along with sufficient intake of essential macro and micronutrients (**Table 5**). This is particularly important in conditions of psychological stress because the stress response substantially increases metabolic demands for nutrients involved in neurotransmitter synthesis, antioxidant defense, and immune regulation. Consequently, poor-quality diets or those lacking essential nutrients may trigger stress over time, leading to heightened and prolonged biological stress responses (288–290). Inflammation and OS are strongly connected to stress pathways, including HPA-axis activity, cortisol dynamics, and micronutrients with redox-enzyme activities and antioxidant potential such as vitamins A/C/D/E/B6/B12, selenium, zinc, iron, copper, folate, magnesium that help in maintaining the redox balance in the microarchitecture in the body which can otherwise enhance downstream stress signaling (289–292). Improving the status of specific nutrients can significantly influence stress physiology. For instance, a randomized controlled study showed that sustained-release vitamin C supplementation (3 g/day for 14 days) significantly reduced salivary cortisol levels, attenuated blood pressure and subjective stress responses following exposure to an acute psychological stressor, the Trier Social Stress Test (TSST), compared with a placebo (293). Similarly, intake of multi-vitamins has been reported to improve cortisol secretion patterns even when subjective measures of perceived stress show inconsistent effects, suggesting the thought of an avid need of nutrient adequacy for a healthy lifestyle as it does interact with stress endocrinology (294).

**Table 5:** Randomized controlled trials (RCTs) in children and adolescents evaluating the effects of nutritional interventions on circulating IL-6 levels.

Clinical trials	Intervention (duration)	Effect on IL-6	References
Adolescents with obesity (~15–16 y)	Omega-3 (n-3 PUFA) vs placebo, crossover (3 months)	Lowered IL-6, TNF-α, IL-1β	(284)
Adolescent girls with obesity	Mediterranean diet vs standard advice (12 weeks)	MD reduced serum IL-6 and hs-CRP vs control.	(261)
Adolescent girls with obesity	Low-GI diet vs healthy recommendation diet (10 weeks)	Low levels of inflammatory markers including IL-6 and hs-CRP	(285)
Children and adolescents with overweight or obesity (6–18 y)	Synbiotic vs placebo (8 weeks)	Decreased IL-6 (and TNF-α); effect partly BMI/weight-dependent.	(286)
Adolescent girls with overweight or obesity	Curcumin 500 mg/day vs placebo + mild diet (10 weeks)	Curcumin reduced IL-6 and improved oxidative stress markers (TAC/MDA).	(287)

This table summarizes key randomized controlled trials conducted in pediatric and adolescent populations that investigated the effects of nutritional interventions on circulating IL-6 concentrations. It outlines study populations, types of interventions, intervention durations, and the main outcomes related to IL-6 levels. Collectively, these studies demonstrate that several nutrition-based strategies can effectively reduce IL-6 levels, and improve other inflammatory markers and oxidative stress indicators, highlighting the potential of dietary interventions in modulating low-grade inflammation during childhood and adolescence.

Nutrient adequacy is also important for low-grade inflammation linked to IL-6 because the type and amount of diet shapes immune tone via various downstream mechanisms (257, 288, 289). High-quality diets that include fruits, vegetables, fibers, whole grains and healthy fats have been shown to contribute to lower inflammatory profile, including reduced IL-6 levels in pediatric populations. In contrast, Western-style diets are commonly associated with high inflammatory markers (257). Applications of the Dietary Inflammatory Index in pediatric and adolescent populations, including youth-adapted versions, have been specially designed to quantify the “inflammatory potential” of diet. These indices have been associated with low-grade inflammatory markers in several studies, reinforcing the notion that the overall dietary pattern and nutrient profile, rather than individual nutrients alone, play a critical role in IL-6 regulation (258–260, 295). Therefore, these findings underscore the importance of nutrient adequacy through healthy, fresh dietary patterns with reduced inflammatory potential to support optimal health, growth, and development in children.

Mechanistically, adequate fiber intake and plant-based diets can attenuate the IL-6 signaling cascade that triggers inflammation and related complications. This occurs through enhanced gut microbial metabolism, such as SCFA production, reinforcing epithelial barrier integrity, and lowering endotoxin-induced immune system activation, which collectively contribute to lower systemic inflammation (289, 296, 297).

Vitamins and trace elements, particularly vitamin D, zinc, selenium, and vitamins C and A, mediate both adaptive and innate immune responses and can mitigate high production of pro-inflammatory cytokines under infection, stress, or metabolic strain (290, 291, 298). Vitamin D induces immunomodulatory effects and is associated both observationally and mechanistically with inflammatory regulatory pathways, including IL-6, which is relevant for chronic inflammation phenotypes. However, supplementation outcomes in pediatric populations may vary depending on underlying conditions, baseline status, and dosage (291, 298).

Nutrient adequacy indirectly influences adiposity. Diets that are energy-dense yet micronutrient-poor can promote visceral fat accumulation, and adipose tissue is a significant source of IL-6 and other inflammatory cytokines. Thus, improvements in dietary quality, even without strict caloric restriction, may reduce IL-6 levels by ameliorating metabolic dysfunction and adipose tissue–related inflammation (257, 289, 299).

Evidence from several randomized dietary pattern trials in youth indicates that improvements in overall diet quality, such as Mediterranean, Dietary Approaches to Stop Hypertension (DASH), and low-glycemic index (GI) dietary patterns and various supplement-based pediatric RCTs (omega-3, synbiotics, curcumin) are associated with reductions in circulating IL-6 levels along with improvements in metabolic or oxidative stress markers (261, 284–287). For instance, an RCT evaluating the effects of a low-GI diet in overweight and adolescent girls with obesity demonstrated significant reductions in inflammatory biomarkers, including high-sensitivity C-reactive protein (hs-CRP) and IL-6, along with increased serum adiponectin levels, indicating improved metabolic and inflammatory regulation (285). Nevertheless, not all nutrient-based interventions reduce inflammatory cytokines such as IL-6. For example, some vitamin D studies showed minimal alterations in systemic inflammation despite improvement in vitamin D status (300). Collectively, these findings suggest that “nutrient adequacy” is more effectively conceptualized as a holistic strategy, encompassing sufficient energy and protein intake, high dietary fiber, and micronutrient-rich foods rather than relying on a single supplement to suppress IL-6 and systemic inflammation (297). In summary, persistent nutrient inadequacy and chronic inflammation during childhood may contribute to long-term health, growth, and developmental consequences.

## 13 Conclusion

In conclusion, psychological and physiological stress-induced IL-6 alters bone remodeling and impairs linear growth in children. While transient IL-6 signaling may support adaptive tissue repair, chronic stress-induced elevation of IL-6 promotes osteoclastogenesis, impairs growth plate chondrogenesis, suppresses GH/IGF-1 signaling, and increases bone marrow adiposity and oxidative stress, the processes that collectively compromise skeletal development.

Future studies should investigate IL-6 in children exposed to chronic stressors, such as academic pressure or child labor, and assess their relationship with skeletal health. Such evidence could help guide public health strategies and policy efforts aimed at reducing environmental stressors that threaten pediatric bone health.
